# Tuning structure and properties of Ti_2_B_2_T_x_ MBenes via halogen terminations

**DOI:** 10.1038/s41699-026-00744-8

**Published:** 2026-10-13

**Authors:** Rodrigo M. Ronchi, Jonas Björk, Johanna Rosen

**Affiliations:** Materials Design Division, Department of Physics, Chemistry, and Biology (IFM), Linköping University, Linköping, Sweden

**Keywords:** Chemistry, Materials science, Physics

## Abstract

MBenes are a relatively new family of two-dimensional (2D) transition metal borides that can be synthesized through selective etching of A-layers from MAB phases. As in MXenes, surface termination is a key factor influencing their properties. Inspired by the recent discovery of Ti_2_B_2_T_x_ MBene, this Density Functional Theory (DFT) study investigates halogen-terminated Ti_2_B_2_T_x_ compounds (T_x_ = Cl, Br, and I) with coverages ranging from 100% (ideal, x = 2) to 50% (x = 1), for which the unoccupied sites are either treated as vacancies or filled with oxygen atoms. Our main findings reveal that oxygen incorporation is favorable for all halogen terminations, and the interlayer binding energies of the multilayer-Ti_2_B_2_T_x_ MBenes are comparable to those of other vdW 2D materials. Further, the MBenes exhibit good mechanical properties and low interlayer sliding energy barriers, suggesting suitability for tribological applications. The effect of the surface termination on the electronic structures and adsorption energies was also investigated for fundamental insights. Finally, the calculated work function ranges from ~3 to 5 eV, indicating that it can be tuned with precise control over termination coverage and composition, offering a powerful route to tailoring MBene properties for improved application-specific performance.

## Introduction

Since the discovery of graphene^1^, research on two-dimensional (2D) materials has expanded rapidly, driven by the unique properties arising from their high surface-area-to-volume ratio and intrinsic low dimensionality. Following graphene, other 2D materials have been discovered, such as boron nitride^2^, transition metal dichalcogenides^3,4^ and, more recently, MXenes^5^. The diverse properties of these 2D materials make them attractive candidates for a wide range of applications, including energy storage, catalysis, gas separation, and water desalination^6–8^.

In 2011, the first compound from a family of layered carbides, nitrides, and carbonitrides, called MAX phases, could be selectively etched to a 2D MXene counterpart^5^. MXenes’ unique properties, combined with the possibility of converting strongly bonded 3D materials to 2D structures, have driven efforts to discover new families of nanolaminated compounds. Among them, increasing attention has been directed towards laminated borides known as MAB phases, which can produce 2D structures composed of metal (M) and boron (B) layers termed MBenes or boridenes^9–12^.

While it is well established that surface terminations play a pivotal role in tuning MXene properties, their influence on MBenes remains largely unexplored. Previous studies have mainly considered O, F, and OH terminations, as these groups can be introduced during the aqueous HF etching and were experimentally reported when Al and Y were etched from (Mo_2/3_Y_1/3_)_2_AlB_2_ using HF acid, resulting in 2D Mo_4/3_B_2-x_^13^. Subsequent efforts to obtain MBenes used a variety of selective etching approaches, including hydrochloric^14–17^ and hydrofluoric acids^13,18,19^, as well as Lewis acids/molten salts^18,20–22^. However, producing stable, freestanding 2D MBene sheets remains challenging, highlighting the greater difficulty in synthesizing MBenes compared to MXenes^11^. Recently, we demonstrated that reacting Ti_2_InB_2_ with ZnCl_2_ enables the synthesis of multilayer Ti_2_B_2_Cl_x_ MBenes, which can be delaminated into single sheets^23,24^.

Here, we use density functional theory (DFT) to systematically investigate how halogen terminations (T_x_ = Cl, Br, and I) and their coverages influence the structural, electronic, and interlayer properties of multilayer and single-sheet Ti_2_B_2_T_x_ MBenes. Based on experimental evidence of mixed halogen/oxygen terminations on MBenes/MXenes surfaces (compiled in ref. ^24^), we explore the effects of oxygen substituting halogen vacancies at non-ideal coverages. Our results reveal that larger halogens (e.g., I) tend to form non-ideal coverages, while oxygen incorporation is energetically favorable, regardless of the halogen species. The interlayer binding energies (7–30 meV/Å²) are comparable to those of other van der Waals 2D materials, and all structures retain metallic character regardless of termination or coverage. Furthermore, their favorable mechanical properties and low sliding barriers suggest promising behavior in tribological applications. Finally, we demonstrate that halogen (and oxygen) terminations can modulate electronic properties and work functions, emphasizing the importance of precise surface chemistry control in MBenes for future research and practical applications.

## Results

The recent synthesis of multilayer Ti_2_B_2_Cl_x_ (x~1.5) along with experimental studies reported in ref. ^24^ suggests that MBenes can exhibit non-ideal surface termination coverages (x < 2) and may contain both halogen and oxygen terminations. This is reminiscent of MXenes, which have reported termination coverages ranging from 100% (fully covered, x = 2) to ≈50% (x ≈ 1)^25^. Understanding how these variations affect structural, electronic, and interlayer properties is crucial.

### ss- and ml-MBenes with ideal termination coverage

**Fig. 1:**
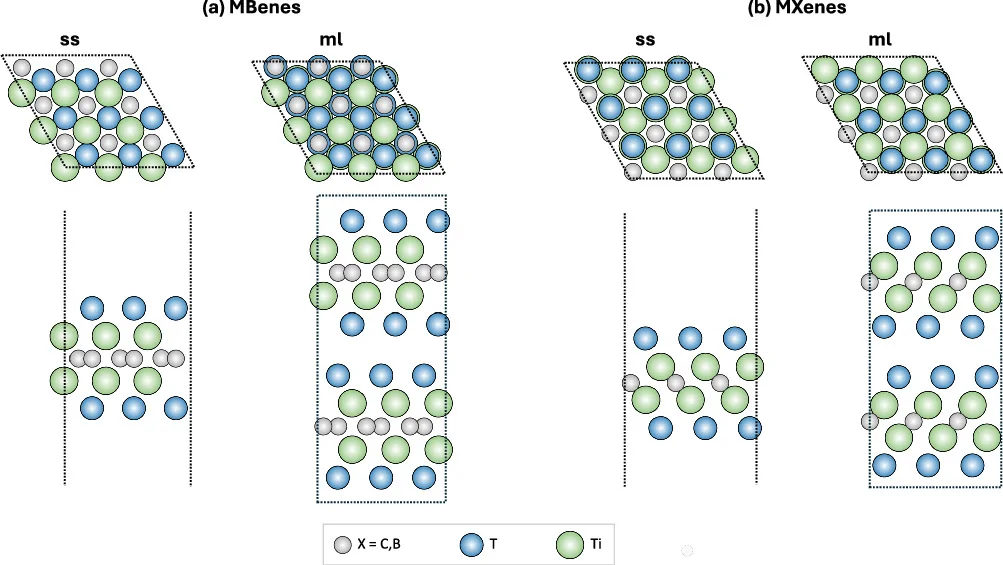
Schematic illustration of the low-energy structures. Top and side views of the single-sheet (ss-) and multilayer (ml-) structures of **a** Ti_2_B_2_T_2_ MBene and **b** Ti_2_CT_2_ MXene.

The ss-Ti_2_B_2_ surface was derived from the hexagonal 3D Ti_2_InB_2_ (*P-6m2*) precursor^19,24^, where boron forms a honeycomb lattice with Ti atoms at its center. Selective etching of In atoms positioned above B_2_ sites results in the Ti_2_B_2_ surface. To identify the most stable termination sites, we mapped the potential energy surface (PES) for Cl, Br, and I on ss-Ti_2_B_2_. Figure S4 shows that all halogens exhibit energy minima above/below the B sites. We then selected four distinct sites to compare the energies of fully terminated ss-Ti_2_B_2_T_x_ materials (shown in Fig. S5). After relaxation, the above-B configuration is energetically favored for all halogens, similar to previous findings for Ti_2_B_2_F_2_ monolayers^26^. This configuration is adopted for subsequent analyses on ss-MBenes and is shown in Fig. 1a.

For fully terminated ml-MBenes (Cl, Br, and I), we computed the PES to evaluate stacking configurations. Figure S6 shows that A-B stacking is the most stable, with two degenerate minima, consistent with STEM characterizations^24^. The flat energy landscape for the ml-case (~5 meV/atom) reflects the weak vdW interactions between layers and suggests easy interlayer sliding, a desirable feature for tribological  applications. The A-B stacked multilayers are used in the following results for ml-MBenes (also represented in Fig. 1a).

Phonon dispersions of fully terminated ss- and ml-structures (Figs. S7 and S8) confirm the dynamical stability (i.e., absence of imaginary modes). Moreover, the materials are also mechanically stable, since their elastic constants satisfy the Born criteria, i.e., ${C}_{11}\, > \,\left|{C}_{12}\right|$, $2{C}_{13}^{2} < \,{C}_{33}({C}_{11}\,+\,{C}_{12})$ and ${C}_{44}\, > \,0$^27–29^. Figure 1b also shows the most stable ss-Ti_2_CT_x_ MXene structure from ref.^25^ (termed 1-FCC_AB_). This compound is used as a benchmark for subsequent discussions owing to the chemical and structural similarities between MBenes and MXenes.

### ml-MBenes with disordered and non-ideal termination coverages

We investigated Ti_2_B_2_T_x_ MBenes with halogen coverages of 100%, 87.5%, 75%, 62.5%, 50% and 0%, corresponding to *x* = 2, 1.75, 1.5, 1.25, 1, and 0, respectively. In addition, for defect formation modeling, we also included a concentration of 98.5% (*x* = 1.97), which represents the removal of only one termination atom from the ml-MBene supercell. To model non-ideal surface coverages, the missing halogen (Ha) atoms were either left vacant (T_*x*_=Ha_*x*_) or replaced by oxygen (T_*x*_ = Ha_*x*_O_2-*x*_). For example, at 75% Cl coverage, these two cases would translate to Ti_2_B_2_Cl_1.5_ and Ti_2_B_2_Cl_1.5_O_0.5_, respectively.

**Fig. 2:**
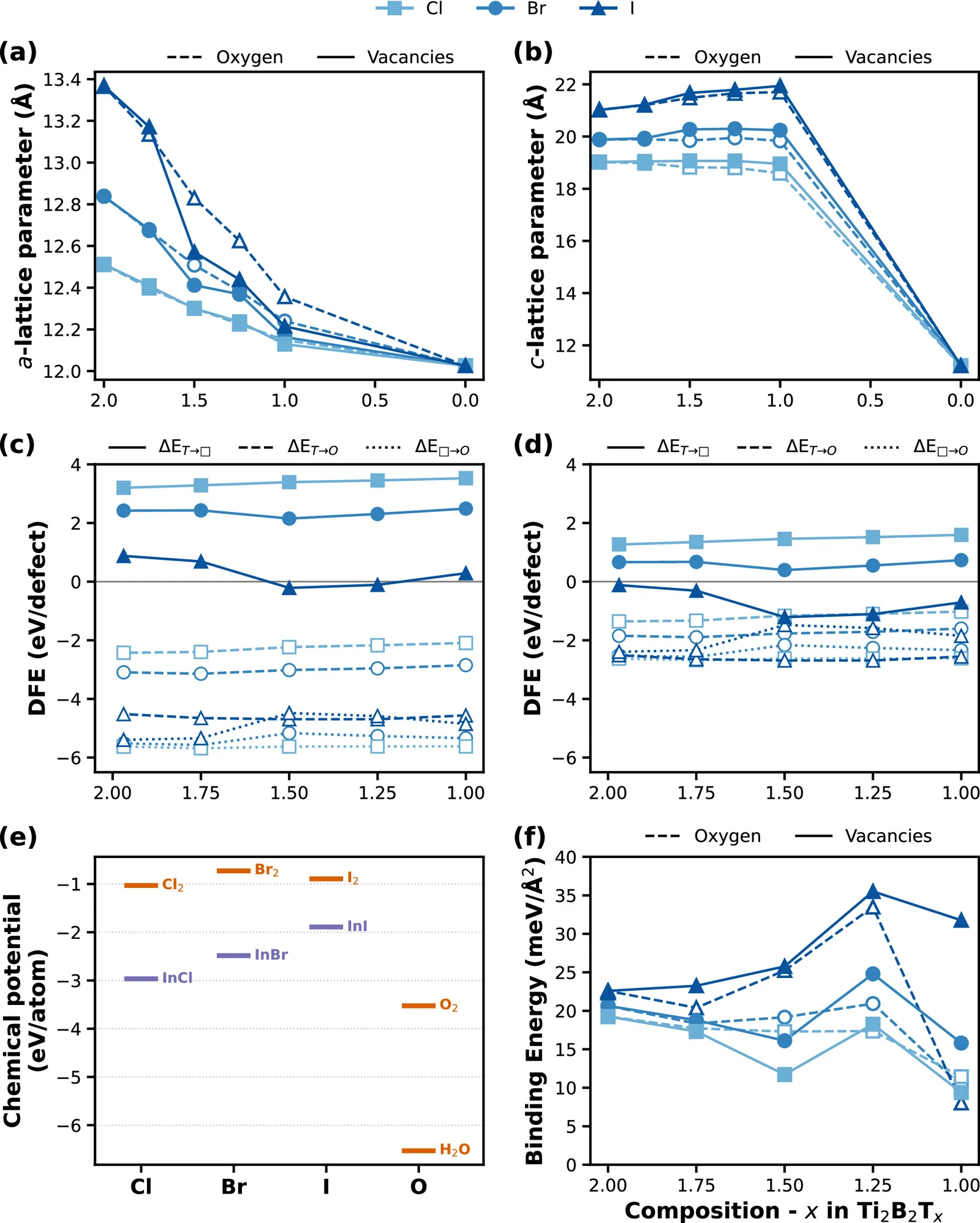
ml-MBenes with different halogen termination coverages, including halogen vacancies and oxygen substitution. **a**, **b** In-plane (a-lattice parameters) and out-of-plane (c-lattice constants) as a function of the termination content and coverage. **c**, **d** Defect formation energies (DFEs), namely the formation energies for creating a halogen vacancy (${\triangle E}_{T\to {{{\square }}}}$), oxygen incorporation into a vacant site (${\Delta E}_{{{{\square }}}\to O}$), and direct substitution of a halogen termination by an oxygen (${\triangle E}_{{{{\rm{T }}}}\to O}$). **c** and **d** use different chemical-potential references: **c**, ground-state elemental phases and **d**, In halides for the halogens and water for oxygen. **e** Chemical potential energies corresponding to the different reference choices. **f** Binding energies, E_b_.

Figure 2a, b show that both the in-plane (*a*) and out-of-plane (*c*) lattice parameters increase along the Cl–Br–I series, which can be correlated with the size of the terminating atoms. Iodine, being larger, requires longer interatomic distances and, consequently, larger lattice parameters compared to bromine and chlorine^25,30^. This effect can also be seen in the charge density difference plots (Fig. S9)^31,32^.

Considering the termination coverage *x*, two opposite trends are observed regardless of whether vacancies or oxygen occupy the mixing sites (though this is more pronounced for the vacant sites and larger termination sizes). While the *a*-lattice parameter decreases with decreasing termination coverage, the *c*-lattice parameter is relatively constant for Cl and increases for Br and I. This indicates a difference between the effects of coverage on strong in-plane bonds, which determine the former, and weak interlayer vdW interactions, related to the latter. In the in-plane case, fewer termination atoms reduce both the in-plane repulsion between the functional groups and the number of strong bonds (e.g., Ti–T_x_ with distances around 2.5–2.8 Å), leading to a decrease in the *a*-lattice constant^25,30^.

The slight increase of the *c*-lattice parameter with the vacancy concentration is likely related to the T_x_–T_x_ vdW interactions being the main attractive component of the interaction between layers. Reducing the number of terminations weakens this attraction, resulting in an expansion of the out-of-plane lattice constant, particularly for larger terminating atoms. Importantly, the absence of abrupt changes in *c*-lattice parameter with halogen coverage indicates that there is no ‘collapse’ into a 3D boride with only one termination layer without van der Waals interactions, as can be seen in Figs. S10-12.

Reducing the termination coverage creates unoccupied termination sites, which can be treated as point defects. The thermodynamic formation barriers for these vacancies, quantified by the defect formation energy (defined in Eqs. 1–3), depend on the choice of chemical potentials μ. Under standard ground-state elemental references (Fig. 2c), all vacancy formation energies (${\Delta E}_{T\to {{{\rm{\square }}}}}$, Eq. 1) are positive, indicating that vacancy creation is endothermic and thermodynamically unfavorable at T = 0 K. Along the Cl–Br–I series, ${\Delta E}_{T\to {{{\rm{\square }}}}}$ decreases (e.g., 3.29, 2.47, and 1.14 eV/defect for x = 1.75), correlating with their increasing halogen atomic radius and decreasing electronegativity. However, evaluating defects against a gaseous baseline (e.g., assuming a chlorine vacancy forms Cl_2_ gas) oversimplifies complex experimental realities.

To better mimic realistic synthesis and post-treatment environments, Fig. 2d shows the defect formation energies using halogen reservoirs bound as indium halides (InCl, InBr, InI), which have been suggested as the most stable halide products^24^, while oxygen was assumed to be supplied via H_2_O during aqueous washing^33^. Under these conditions, our main conclusions remain qualitatively similar, though with quantitative refinements. Notably, the thermodynamic cost of defect formation approaches zero across all systems. This shift occurs because the highly reactive elemental gas references (Cl_2_ and O_2_) represent an upper bound for the chemical potential, whereas halogens bound to a metal surface or oxygen bound in water represent significantly more stable, lower-energy reservoirs. Consequently, the formation of –I vacancies becomes exergonic (${\Delta E}_{T\to {{{\rm{\square }}}}} < 0$), suggesting that iodine-terminated surfaces are inherently prone to spontaneous vacancy formation. When oxygen subsequently occupies these vacant sites, ${\Delta E}_{{{{\rm{\square }}}}\to O}\,$(Eq. 2) remains negative across all halogen types. Although thermodynamically driven even under mild, moisture-rich conditions, oxidation shows a reduced driving force compared to exposure to oxygen gas. Finally, the direct substitution energy ${\Delta E}_{T\to O}$ (Eq. 3) remains energetically favorable but higher than ${\Delta E}_{{{{\rm{\square }}}}\to O}$, reinforcing that oxygen incorporation is easier in pre-existing vacancies compared to the direct replacement of functional groups on fully terminated Ti_2_B_2_T_2_ surfaces.

Figure 2d shows the interlayer binding energy, as defined in Eq. 4, which represents the energy required to break the multilayer material into single sheets. In general, E_b_ ranges from 7 to 30 meV/Å^2^, comparable to calculated binding energies of halogen-terminated MXenes, graphite, hexagonal boron nitride, and transition-metal dichalcogenides (10–40 meV/Å^2^ with different functionals)^25,34–36^. In particular, the binding energies of graphite, h-BN and MoS_2_ calculated with the same exchange-correlation functional were 22.7, 20.9, and 23.5 meV/Å^2^^36^. The fully terminated Ti_2_CT_x_ MXenes (18.0, 19.5, and 21 meV/Å^2^ for Cl, Br, and I terminations) have very similar E_b_ values compared to MBenes (19.3, 20.6, and 22.3 meV/Å^2^, respectively). The interlayer binding energy parameter correlates with the mechanical exfoliation potential, and ml-materials with low E_b_ may be more easily converted into single sheets.

### ss-MBenes with disordered and non-ideal termination coverages

As depicted in Fig. S13a, the *a*-lattice parameter decreases continuously with decreasing termination coverage *x*, which becomes more pronounced for larger terminating species *T*. This behavior occurs irrespective of whether the termination sites are vacant or occupied by oxygen, though the decrease is substantially greater in the vacancy case. The difference in lattice parameters between having vacancies and substituting oxygen also increases with termination size. Small terminating atoms induce minor structural changes regardless of site occupancy, whereas larger terminations have a strong impact on the lattice parameters. For fully terminated structures, MBenes and MXenes display comparable increases in lattice parameters: Br- and I-terminated MBenes (MXenes) show increases of 4.7% and 10.9% (5.2% and 11.6%), respectively, relative to the Cl-terminated version. The structural similarity between ss- and ml-MBenes is illustrated in Fig. S13b, which shows that their *a*-lattice parameters differ by only ~0.5%. This similarity, together with the electronic structure analysis presented below and in prior MXene studies^25^, indicates that the weak van der Waals interlayer interactions have only a minor influence on the multilayer structures.

MBenes have potential for diverse applications, including energy storage, conversion technologies, and tribology. To evaluate this broad application potential, DFT calculations were used to screen key foundational properties, such as interlayer sliding energy barriers, work functions, and Li-, Na-, and Zn- ion adsorption. While these metrics provide valuable preliminary indicators for tribology, photovoltaics, and energy storage, they are intended here only as an initial computational screening to highlight promising avenues for more detailed, application-specific evaluation and testing.

As a preliminary assessment of the finite-temperature stability of the mixed and partially terminated structures, short *ab initio* molecular dynamics (AIMD) simulations were performed at 300 K for selected representative structures (Fig. S23–S25). The Cl/O-terminated MXenes, with 37.5% of the Cl terminations replaced by O, remained structurally stable throughout the simulations, with no apparent termination migration, clustering, or significant changes in the layer structure. For the partially I-terminated MXenes, the structure with 50% I coverage likewise retained all I atoms at the surface, whereas some I atoms detached from the surface in the structure with 75% I coverage. These observations are consistent with our previous results on MXenes indicating that an I coverage of approximately 50% is energetically preferred, in contrast to the full Br and Cl coverages^37^. While these short AIMD simulations provide an initial indication of the finite-temperature stability of the considered structures, a more comprehensive investigation over a broader range of compositions, coverages, and simulation times would be required to fully assess their finite-temperature structural stability.

### Electronic properties of ml- and ss-MBenes

**Fig. 3:**
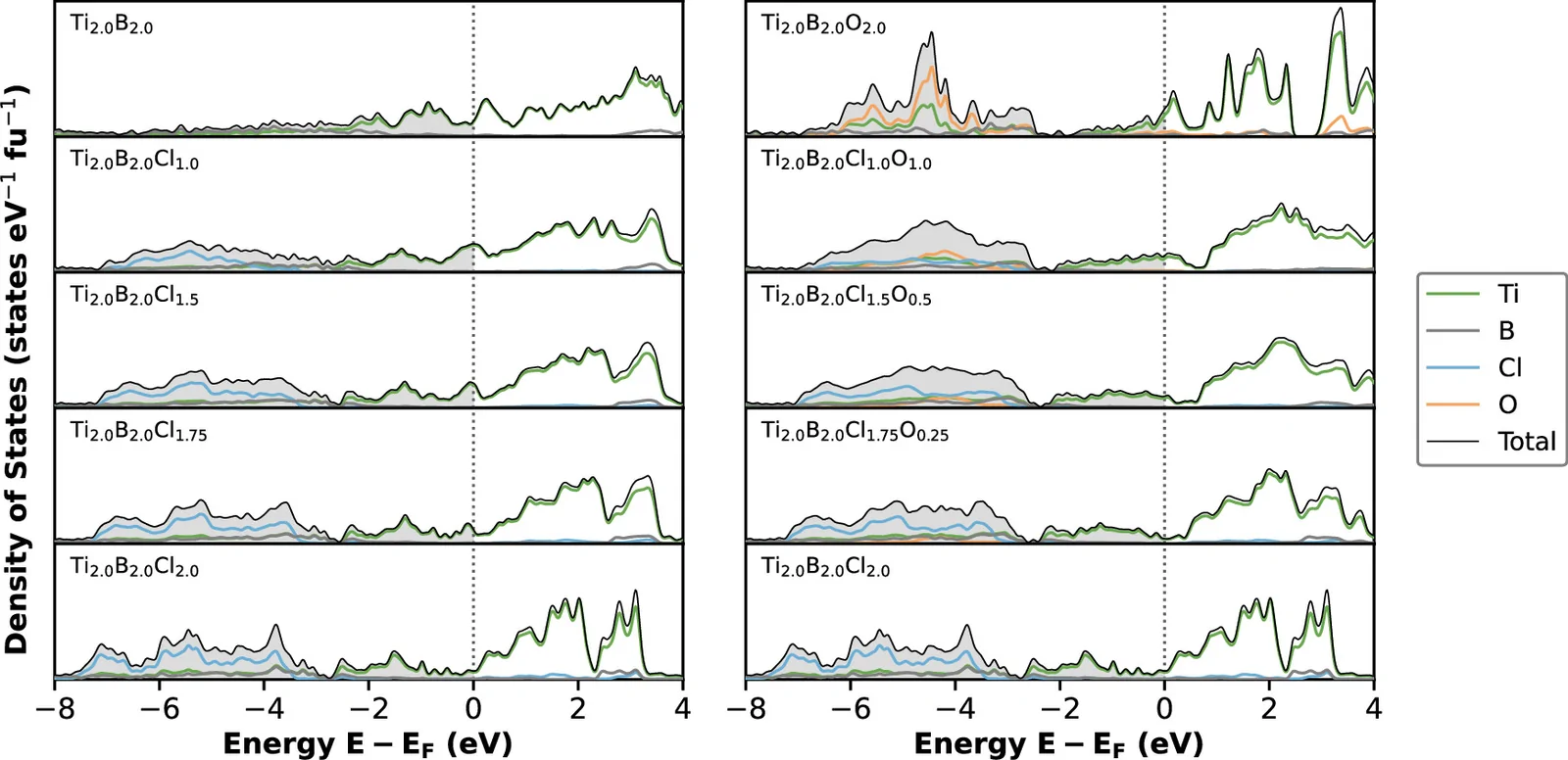
Density of states of Cl-terminated ml-MBenes with different halogen coverages, with and without oxygen substitution. The DOS (y-axis) is normalized per formula unit (f.u.). The shaded regions indicate the occupied electronic states below the Fermi level (E_F_).

The electronic properties of ml-MBenes reflect their weak interlayer bonding, as evidenced by nearly indistinguishable densities of states (DOS) for ss- and ml-systems (Fig. S14). The termination-dependent effects on the electronic properties were examined. Figure 3 shows representative DOS for Ti_2_B_2_Cl_x_O_2-x_ ml-MBenes (with additional data for other systems shown in Figs. S15–S20). All systems exhibit metallic behavior independent of halogen species, termination coverage, or oxygen presence, aligning with experimental Ti_2_B_2_Cl_x_^24^ and MXenes^25,38^ data.

In general, the electronic structure of the MBenes can be grouped into three main regions: (1) Hybridized metal-boron-termination (and oxygen if present) states between −8 and −2 eV; (2) Metal-dominated states between −2 and 0 eV; (3) A majority of metal states with minor contributions from halogen and/or oxygen atoms possibly appearing above the Fermi energy. Notably, the number of states at the Fermi energy slightly increases with reduced termination coverage, regardless of vacant sites or oxygen incorporation. This can be attributed to the presence of fewer M-T bonds, giving rise to an increased number of non-bonding metal states. This trend, previously seen for MXenes^25^, suggests enhanced electrical conductivity in non-ideal MBenes.

### Work function of ss-MBenes

**Fig. 4:**
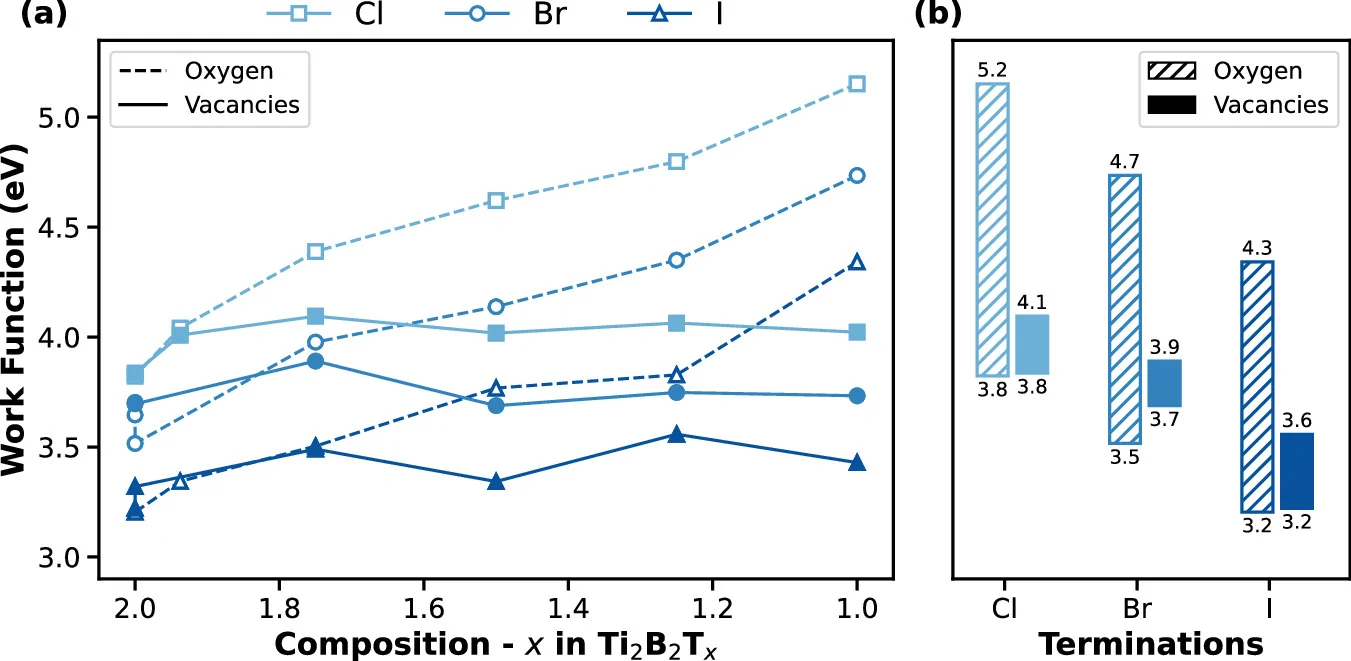
Work function of ss-MBenes with disordered and non-ideal termination coverages. **a** Work function as a function of termination type and coverage. **b** Distribution of work-function values for each halogen-terminated MBene, depending on if the missing sites are left as vacancies or populated with oxygen.

Closely related to the changes in the electronic properties, the work function is an important parameter for electronic and photovoltaic applications. It represents the minimum energy required to extract an electron from a material, calculated as the energy difference between the vacuum potential and Fermi level. The calculated work function of the ss-MBenes shown in Fig. 4 ranges from ~3 to 5 eV. Specifically, ss-Ti_2_B_2_Cl_2_ exhibits a work function of 3.8 eV, lower than the previously reported value for a 2D single-sheet Ti_2_B_2_F_2_ structure (~5 eV)^26^, implying a trend of F > Cl > Br > I. This likely results from reduced surface dipole moments^39^, supported by reduced Ti–T_x_ electron transfer for larger halogens (Table S1 and Fig. S21b).

The presence of vacant sites or oxygen terminations has a distinct influence: vacancies have a minor impact (e.g., only 0.15 eV variation in Ti_2_B_2_Br_x_), while the insertion of oxygen consistently increases the work function. This increase is due to the higher work function of oxygen-terminated MBenes^26^ and MXenes^39^ compared to those with fluorine, –OH, or other terminations.

These findings demonstrate that controlled surface coverage, particularly through mixed halogen/oxygen termination, enables work function tuning. This flexibility is a key advantage of 2D materials, which enables optimizing energy-level alignment for applications such as electron emitters, Schottky barriers, and charge transport layers in photovoltaic technology^39,40^.

### Mechanical properties of ml- and ss-MBenes

The mechanical response of 2D materials can be characterized through different fundamental properties. Here, we will focus on Young's modulus (*E*), which quantifies stiffness, i.e. the material's resistance to elastic deformation, and the shear modulus (*G*), which describes its resistance to shear deformation (where a larger shear modulus indicates a higher rigidity of the material against shape distortion under tangential forces). These properties are derived from the elastic constants (*c*_*ij*_), which can be reliably estimated using DFT.

**Fig. 5:**
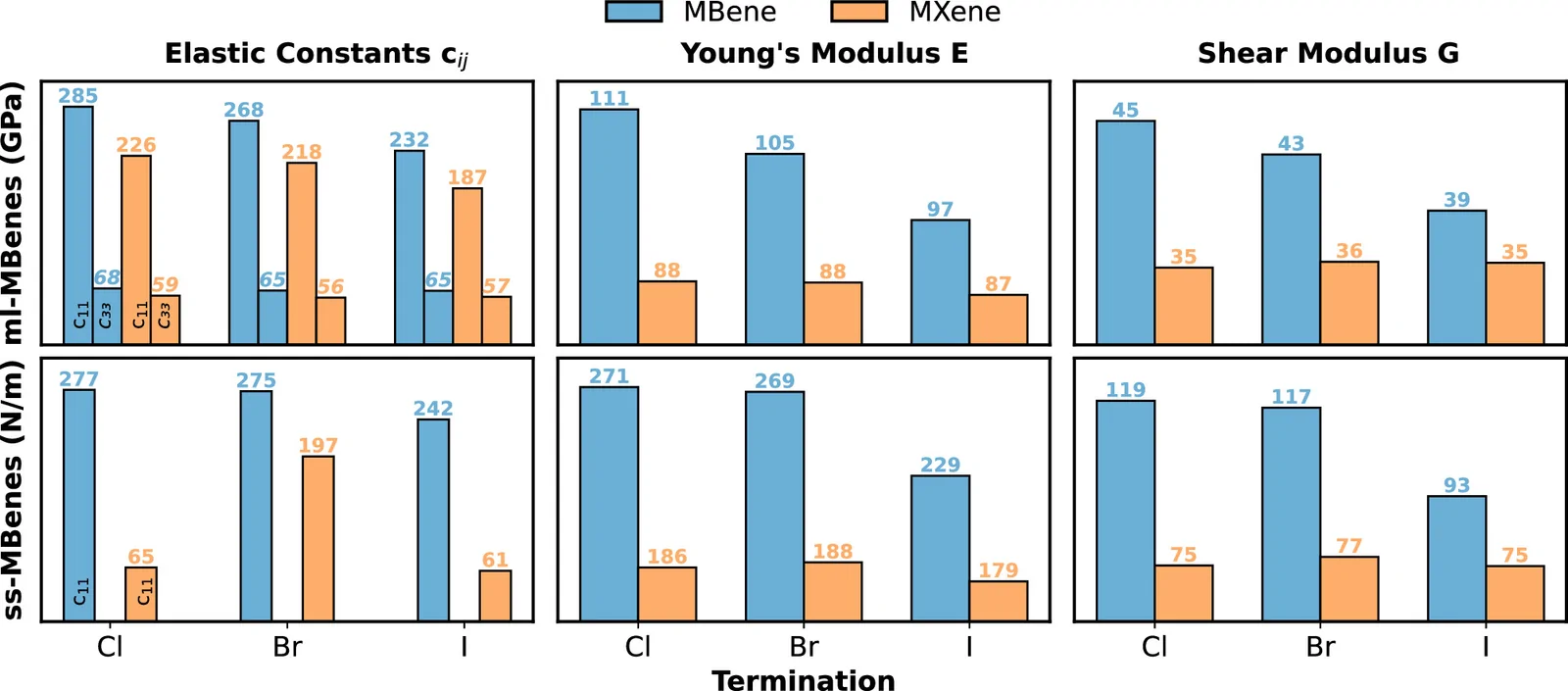
Mechanical properties of ideal MBenes. DFT-calculated mechanical properties of ss- and ml-MBenes compared with MXenes. Mechanical properties are reported in N/m for ss-compounds and GPa for the multilayers.

Figure 5 presents the calculated mechanical properties for MBenes (note that the different units between ml- and ss-examples prevent a direct comparison). The significant difference between the in-plane *c*_*11*_ and the out-of-plane *c*_*33*_ elastic constants reveals strong anisotropy (*c*_*33*_ is lower than half of *c*_*11*_), as a direct consequence of the different bonding. The anisotropic behavior originates from the in-plane properties being governed by a mixture of strong ionic, metallic, and covalent bonds, such as Ti–T_x_ and B–B bonds, while weak van der Waals interactions between the terminations are the main contributors in the out-of-plane direction.

Both ss- and ml-MBenes follow E_Cl_ > E_Br_ > E_I_, correlating with larger termination sizes and lower electronegativity values along the halogen series. Chemical bonding analysis (Tables S1, S2 and Fig. S21) confirms this trend, showing decreased ionic (via Lowedin/Bader) and covalent (via -COHP) contributions. More specifically, Lowedin and Bader charges show a decrease in Ti–T_x_ charge transfer (and consequently, ionic bonding strength), while the integrated -COHP values decrease for B–B and Ti–B bonds (i.e., reflecting reduced covalent strength).

Importantly, while the mechanical properties of ml-MBenes show continuous trends with respect to the halogen terminations, ss-MBenes exhibit similar E values for Cl/Br terminations with significant reduction for I. The major difference between the ml- and ss-case arises from the charge redistribution of the whole structure when the weak interlayer interactions between terminations are not present. This can be seen in the slight difference of Bader/Lowedin charges from ml to ss-compounds. For the latter, Ti–T_x_ charge transfer and B-B iCOHP from Cl and Br are similar, with a significant decrease for I. Significant differences between F and Cl in bonding features are not expected, since the results reported here are particularly close to the TiBF system reported in ref.^26^.

Compared to other 2D materials, the MBenes demonstrate intermediate mechanical properties. Their Young’s moduli fall below that of graphene (≈1000 GPa) but within the range of graphene oxide (200–300 GPa), MXenes (150–300 GPa), and hexagonal boron nitride (250–280 GPa)^41,42^. Remarkably, ss-MBenes show a 28–45% higher Young’s modulus than Ti_2_CT_x_ MXenes with the same surface terminations, likely due to the strong in-plane covalent B–B bonds, comparable to M–C and/or M–N bonds in MAX phases^43^.

### Interlayer sliding of ml-MBenes

In addition to the lubricant properties of the 3D MAB-phase precursors^10^, the weakly bonded multilayer structure combined with high mechanical strength renders MBenes as promising candidates for solid lubricant applications. We quantified the intrinsic sliding resistance (related to the coefficient of friction) through PES calculations on ml-MBenes, where the energy barrier ($\Delta E={E}_{\max }-{E}_{\min }$) characterizes the corrugation landscape^44–46^. Using a 7×7 grid of relative layer positions with fixed volume and interlayer distance, we evaluated the PES under zero load conditions. Note that this is slightly different from the PES approach discussed in the Methods section, since the volume/interlayer distances were also kept fixed here.

**Fig. 6:**
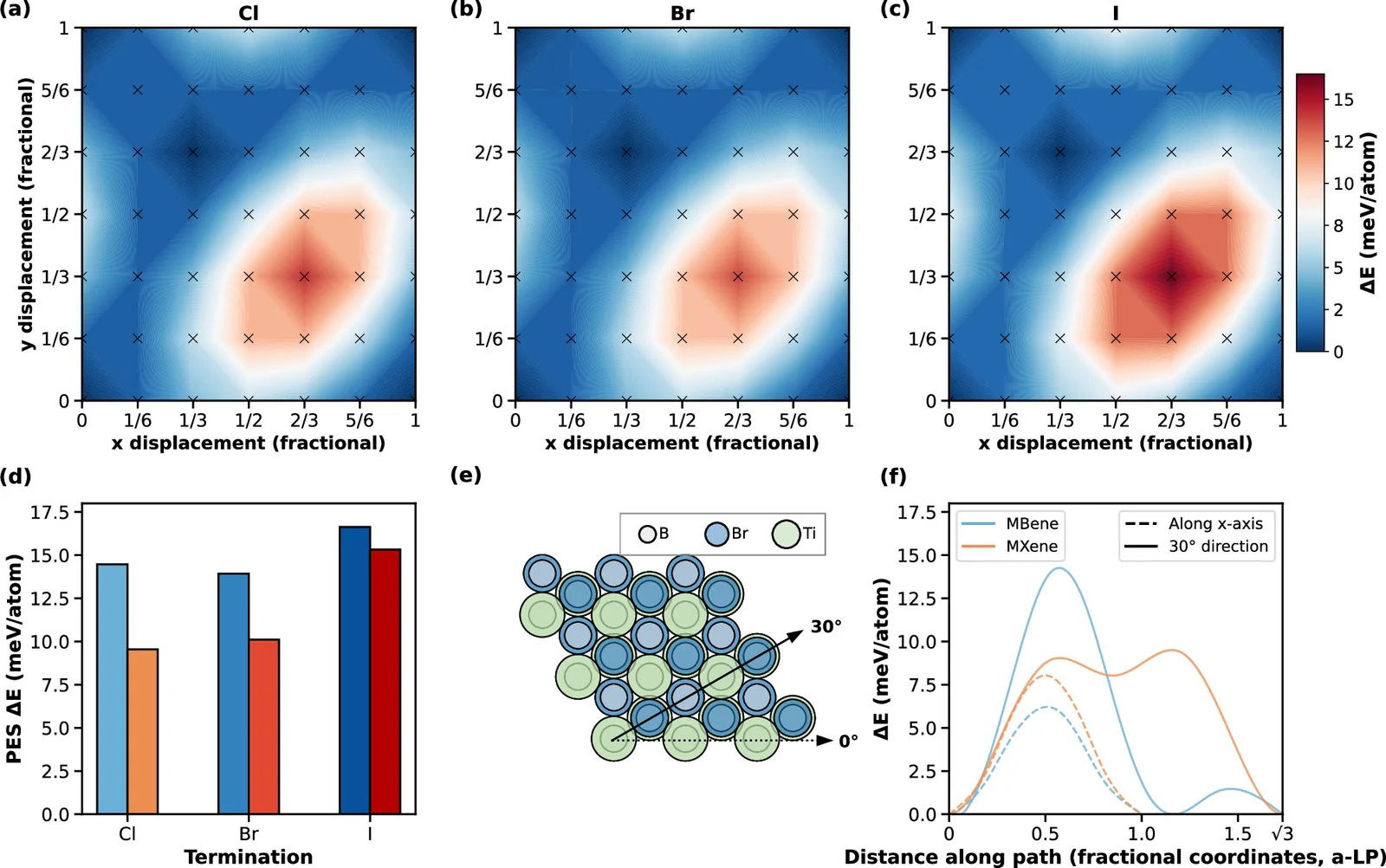
Interlayer sliding properties of ml-MBenes. **a**–**c** Potential energy surfaces (PESs) of (1×1×1) Ti_2_B_2_T_2_ MBenes with T = Cl, Br, and I. All atomic positions, lattice vectors, and cell volume were kept fixed during the single-point calculations. **d** Maximum sliding energy barrier of MBenes and Ti_2_CT_x_ MXenes obtained from the PESs. **e** Top view of the MBene structure illustrating the two sliding directions. **f** Sliding energy barriers of Cl-terminated MBenes (orange lines) and Cl-terminated MXenes (blue lines) along the two sliding paths.

The calculated PES for the ml-Ti_2_B_2_T_2_ MBenes and Ti_2_CT_2_ MXenes (with ideal coverages) are shown in Fig. 6a–c and Fig. S22, respectively, with the maximum energy barriers ΔE compiled in Fig. 6d. The energy barriers follow the trend I > Br ≈ Cl, inversely correlating with the mechanical properties (particularly the shear modulus, as sliding is directly related to shear resistance). This may be attributed to the trends found in size (I > Br > Cl), binding energy (I > Br > Cl), and bond strength (I < Br < Cl), which increase the interlayer resistance to sliding.

Although Cl- and Br-terminated MBenes exhibit slightly higher ΔE than corresponding MXenes, they present distinct anisotropic sliding behavior. The PES topology shows that the MBene barrier is concentrated in a specific x- and y-displacement region, contrasting with MXenes’ symmetric barrier distribution. This was further verified by comparing two different sliding paths (0- and 30-degree orientations) depicted in Fig. 6e. As can be seen in Fig. 6f, MXenes have low orientation dependence, since they maintain nearly constant barriers (8–9 meV/atom) irrespective of slip paths. Conversely, MBenes show greater variation depending on the slip path (6–14 meV/atom), with reduced resistance at 30°. Thus, considering multiple flakes and different force directions in practical applications, the different sliding paths in MBenes may contribute to lowering the overall energy barrier.

The energy barriers of MBenes are in the range of common lubricants and other 2D materials, including MoS_2_ bilayer (9–15 meV/atom)^47^ and multilayer graphite (around 14 meV/atom)^31,48^. Although the tribological performance of MBenes has rarely been investigated^10^, the combination of a weakly bonded multilayer structure, high mechanical strength, and maximum barrier sliding behavior similar to other 2D lubricants may enable their use in tribological applications. They could be used as solid lubricants or in heterogeneous interfaces with other 2D materials, an approach that has been found to significantly decrease ΔE of each isolated component in graphene and MoS_2_^44^.

### Li-, Na-, and Zn- ion adsorption of ss-MBenes

While theoretical studies have suggested an ion storage potential for bare MBenes^49,50^, practical applications must consider the terminations arising from the selective etching strategies (or other exfoliation methods). Experimental characterization of HfBO showed reversible capacities from 244 to 125 mAhg^−^^1^ at current densities from 0.05 to 1 Ag^−1^^18^, while halogenated MXenes have been considered promising for Zn-ion batteries^51^. It is important to highlight that studies comparing different terminations (especially for halogens) of MBenes and MXenes in battery applications have been relatively scarce.

**Fig. 7:**
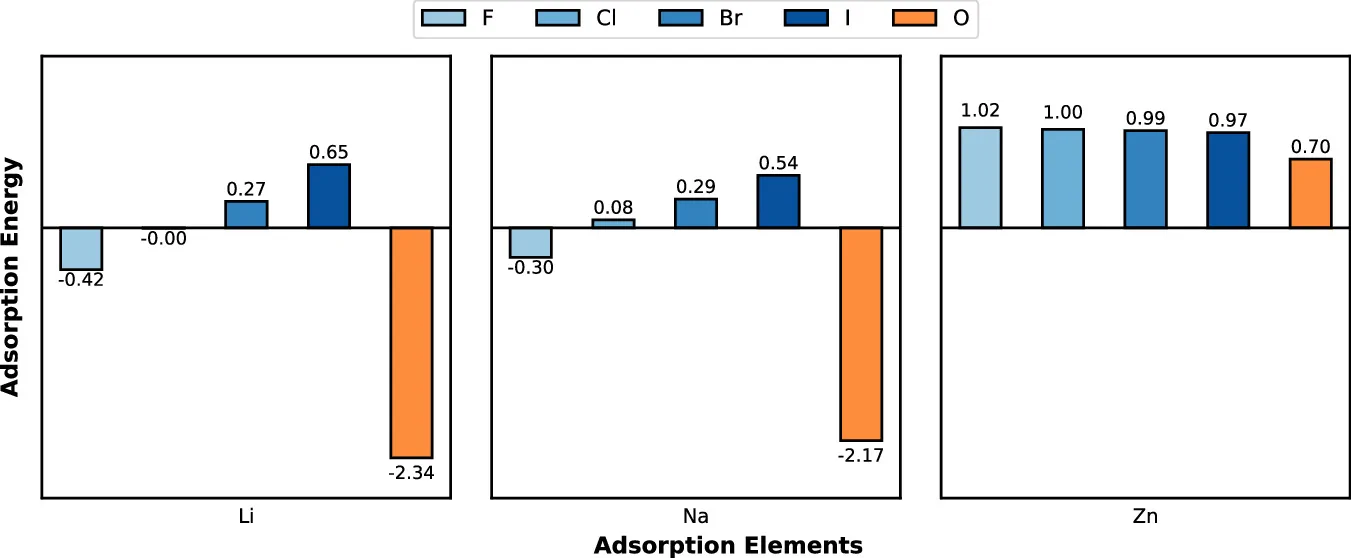
Adsorption energies of Li, Na, and Zn on ideally terminated ss-Ti_2_B_2_T_2_ MBenes. Adsorption energies of Li, Na, and Zn on ideal ss-Ti_2_B_2_T_2_ MBenes with T = F, Cl, Br, I, and O. Results for each termination are color-coded according to the legend. Panels correspond to the respective adsorbates, as indicated by the labels.

To provide more comprehensive insights, we perform an initial screening of halogen-terminated MBenes, by evaluating the adsorption of a single Li, Na, or Zn atom on three high-symmetry adsorption sites of a 3×3 ss-Ti_2_B_2_T_2_ supercell (T = F, Cl, Br, I, and O). For each site, the adsorption energy ${E}_{{ads}}$ per atom was calculated using Eq. 5. Figure 7 shows the adsorption energies for the most stable configuration. Most systems exhibit a positive ${E}_{{ads}}$, which also becomes less favorable along the row of halogens, especially for the adsorption of Li and Na ions.

These findings are similar to the adsorption energy trend O > F > Cl > Br verified previously in Ti_3_C_2_T_2_ ss-MXenes^52^, attributed to the increased electronegativity of the terminations, enhancing the ionic character of the bond between T and Li, resulting in more negative ${E}_{{ads}}$. Furthermore, the preference for oxygen instead of fluorine terminations aligns with theoretical^53–55^ and experimental^56,57^ MXene literature. In fact, thermal annealing post-processing usually improves MXene anode performance in LIBs due to the removal of undesirable –OH and –F terminations. More recently, the delaminated Ti_2_B_2_T_z_ structures showed improved Li-ion battery performance compared to their multilayer counterparts, which was attributed to the increase in surface area and enhanced oxidation of the flakes (i.e., an increase in the oxygen content and decrease in Cl)^23^.

## Discussion

In summary, our systematic DFT study of Ti_2_B_2_T_x_ MBenes (T = Cl, Br, I) provides fundamental insights into the effects of halogen terminations, their coverage, and oxygen incorporation on their structural and functional properties. Multilayer and single-sheet MBenes exhibit nearly identical trends due to weak van der Waals interlayer interactions. Their range of binding energies, comparable to those of other vdW 2D materials, suggests that monolayers can be obtained via exfoliation. Structurally, larger termination species (such as iodine) are more prone to non-ideal surface coverage, and the incorporation of oxygen into vacant termination sites was found to be energetically favorable for all halogen terminations. The electronic structure shows metallic behavior, and the ml-MBenes display elastic constants between 230 and 285 GPa depending on the surface termination, values that fall within the ranges reported for graphene oxide, MXenes, and hexagonal boron nitride. The combination of good mechanical properties and low sliding energy barriers, comparable to those of established lubricants (e.g., MoS_2_ and graphite), suggests their potential for tribological applications. Further, the adsorption energies of Li, Na, and Zn atoms were evaluated to assess their potential for energy storage, indicating a preference for oxygen terminations. The terminations were also shown to have a significant role in determining the electronic properties and work functions of these compounds. Altogether, controlling the surface chemistry of MBenes allows for the tailoring of their electronic properties and work functions (ranging from ~3 to 5 eV), which is essential for future research and diverse applications, such as energy storage and photovoltaics.

## Methods

### Structural relaxation

All DFT^58,59^ calculations were performed using the Vienna Ab initio Simulation Package (VASP)^60,61^, with electron-ion interactions treated by the projector augmented wave (PAW) method^62,63^, and using a plane-wave basis set expanded to a cutoff energy of 520 eV. The rev-vdW-DF2 functional^36^ was employed to describe exchange-correlation interactions, which has been shown to accurately reproduce the structural parameters of multilayer MXenes^64^ as well as both the lattice parameters and interlayer binding energy of graphite^36,65^. Structural relaxations used Monkhorst–Pack k-point grids^66^ with a density of 1/6 Å^-1^. The self-consistency criteria for energy and forces were set to 10^−6^ eV/atom and 10^−2^ eV/Å, respectively. For single-sheet MBene calculations, a vacuum layer of about 20 Å was applied to avoid spurious interactions from the periodic images.

### Adsorption sites and stacking analysis

The preferred termination sites on Ti_2_B_2_ single-sheet MBene (ss-MBene) were identified by computing the potential energy surface (PES) of two termination atoms in a 2 × 2 supercell. The termination atoms were placed on opposite sides of the MBene layer (i.e., one above and one below the M–B sheet) and systematically sampled on a 7 × 7 xy-grid, with fractional coordinates restricted to 0–0.5 due to symmetry.

Furthermore, four high-symmetry sites were chosen to compare the energies of fully terminated ss-Ti_2_B_2_T_x_: (i) above B (hollow Ti sites), (ii) above Ti, (iii) center (above B-B honeycomb), and (iv) asymmetric (one termination above site B_1_ and another below site B_2_).

A PES was also used to assess the different possible stacking configurations of fully terminated multilayer MBene (ml-MBene). In this case, the unit cell consisted of two MBene sheets, in which the top layer was shifted along the fractional coordinates (from 0 to 1), while the bottom layer remained in its original position. During relaxation, only atomic positions along the z-axis and cell volumes were optimized, while *x*- and *y*-coordinates remained fixed.

### Phonon and dynamical stability analysis

For the dynamical stability analysis, we reconverged the unit cell with stricter parameters: a k-point density of 0.15 Å^-1^ and convergence criteria of 10^-7^ eV/atom (energy) and 10^-3^ eV/Å (forces). Phonon dispersion relations were obtained using Phonopy^67,68^ on supercells with each lattice parameter ≥10 Å. Force constants were constrained to preserve rotational/translational invariance by enforcing the Born-Huang conditions^29^ via the hiPhive code^69^, which corrects artificial imaginary frequencies near the Γ-point arising from numerical issues in 2D materials^34,70^.

### Chemical bonding and charge analysis

Chemical bonding was investigated using the LOBSTER program v5.0.0^71^ to generate projected Crystal Orbital Hamiltonian Populations (pCOHP)^72–74^. Results are presented as −pCOHP, where positive (negative) values indicate bonding (antibonding) interactions and zero corresponds to nonbonding states. Bonding strength was quantified by integrating the occupied states of the −pCOHP curves (−IpCOHP) for first-neighbor interactions, with averaged values reported (i.e., the IpCOHP contribution of a certain bond divided by the number of interactions identified). Löwdin charges were obtained from LOBSTER, while Bader charges^75,76^ were computed separately. The mechanical properties of bulk polycrystals were estimated via Hill’s theory^77^, using the energy-strain method in VASPKIT^78^. The charge difference information was extracted with VASPKIT, and the images were generated using VESTA^79^.

### Modeling disordered terminations and non-ideal coverages

Disordered (T_x_ composed of mixed O and halogen atoms, x = 2) and non-ideal (vacancies, x < 2) termination distributions were modeled using special quasi-random structures (SQS)^80^ generated using the *mcsqs* function available in the ATAT code^81,82^. Based on their SQS (Fig. S1) and DFT results via energy and structural convergence tests (Fig. S2), 96-atom (4×4×1) SQS supercells were used for ss-MBenes. For ml-MBenes, to maintain the number of configurations in each layer, we doubled the number of atoms to 192 by constructing a 4 × 4 × 2 supercell. The k-point density and convergence parameters were optimized to ensure energy differences below 3 meV/atom. This threshold was estimated by comparing the energies and lattice parameters across three SQS cells of the same composition (see Fig. S2b).

### Interlayer binding energy and defect formation energy

The defect formation energy for vacant termination sites was computed as:

1 $${\Delta E}_{{{{\rm{T}}}}\to \square}=\frac{E\left({{Ti}}_{2}{B}_{2}{T}_{x}\right)-E\left({{Ti}}_{2}{B}_{2}{T}_{2}\right)+\,{\mu }_{i}{N}_{i}}{{N}_{i}}$$

where $E\left({{Ti}}_{2}{B}_{2}{T}_{x}\right)$ and $E\left({{Ti}}_{2}{B}_{2}{T}_{2}\right)$ are the energies of non-ideal (x < 2) and ideal (x = 2) termination coverages. ${N}_{i}$ is the number of termination species removed from the ideal supercell, and ${\mu }_{i}$ is the chemical potential of species i, taken as the energy per atom of its ground-state elemental structure.

For oxygen-substituted vacancies, two reference states were considered:

1. Relative to non-ideal MBene (Ti_2_B_2_T_x_), meaning the defect formation energy of an oxygen adsorption into a vacant site:
  2 $${\Delta E}_{{{{\square}}}\to O}=\frac{E\left({{Ti}}_{2}{B}_{2}{T}_{x}{O}_{2-x}\right)-E\left({{Ti}}_{2}{B}_{2}{T}_{x}\right)-{\mu }_{O}{N}_{i}}{{N}_{i}}$$
2. Relative to ideal termination (Ti_2_B_2_T_2_), representing the defect formation energy of a termination substitution:

3 $${\Delta E}_{{{{\rm{T}}}}\to O}=\frac{E\left({{Ti}}_{2}{B}_{2}{T}_{x}{O}_{2-x}\right)-E\left({{Ti}}_{2}{B}_{2}{T}_{2}\right)+\,{\mu }_{T}{N}_{i}-{\mu }_{O}{N}_{i}}{{N}_{i}}$$

where $E\left({{Ti}}_{2}{B}_{2}{T}_{x}{O}_{2-x}\right)$, $E\left({{Ti}}_{2}{B}_{2}{T}_{2}\right)$ and $E\left({{Ti}}_{2}{B}_{2}{T}_{x}\right)$ are the total energies of the supercells with and without oxygen at ideal and non-ideal termination coverages. ${\mu }_{T}$ and ${\mu }_{O}$ are the chemical potentials of the termination species and oxygen, respectively, taken as the energy per atom of its ground-state elemental phase (unless specified otherwise in the text). ${N}_{i}$ represents the number of termination species removed (for T) or added (for O).

The interlayer binding energy ${E}_{b}$, representing the energy difference between ml-MBene (3D) and ss-MBene (2D), was calculated as:

4 $${E}_{b}=\frac{2{E}_{2D}^{* }-\,{E}_{3D}}{2A}$$

where ${E}_{3D}$ and ${E}_{2D}^{* }$ are the total energies of the relaxed ml- and ss-MBenes while A is the ml-MBene surface area. Two different approaches were used, which differed by ${E}_{2D}^{* }$: (i) “constrained”, allowing only atomic positions to be relaxed (fixed unit cell parameters) and (ii) “unconstrained”, optimizing all degrees of freedom (including lattice parameters). Both yielded similar results, because the surface area of ml-and ss-MBenes is approximately the same (see Fig. S3).

### Li-, Na- and Zn- ion adsorption

The adsorption energy ${E}_{{ads}}$ per atom was calculated as:

5 $${E}_{{ads}}=\frac{E\left({MBene}+{ads}\right)-{nE}\left({MBene}\right)-{\mu }_{{ads}}}{n}$$

where $E\left({MBene}+{ads}\right)$ and $E\left({MBene}\right)$ are the total energies of the supercell with and without the adsorbed atom, respectively. ${\mu }_{{ads}}$ refers to the total energy of a single Li, Na, or Zn atom in its metallic elemental ground-state structure, and n is the number of adsorbed atoms.

## Supplementary information

SI_FINAL

## Data Availability

The authors declare that the data supporting the findings are available within the paper and its Supplementary Information. The corresponding authors can also provide data upon reasonable request.

## Code availability

The SQS supercells were generated using ATAT^80,81^, DFT calculations were performed using VASP^59,60^, and post-processing was carried out using VASPKIT^77^ and LOBSTER (v5.0.0)^70^. The Python scripts used for data analysis, visualization, and figure generation are specific to the data structures and analysis workflows of this study.
