# Supplementary material for: Tuning structure and properties of Ti2B2Tx MBenes via halogen terminations
Source: NPJ 2D Mater Appl. 2026 Oct 13;10(1):83. doi: 10.1038/s41699-026-00744-8 (PMC13661243; doi:10.1038/s41699-026-00744-8)
Supplement: Supplementary file 1 [file 41699_2026_744_MOESM1_ESM.pdf]

The effect of halogen surface termination coverage into $Ti_2B_2T_x$ MBenes structure, stability, electronic and mechanical properties

Rodrigo M. Ronchi [1], Jonas Björk [1], Johanna Rosen [1]

[1] Materials Design division, Department of Physics, Chemistry, and Biology (IFM), Linköping University, SE-581 83 Linköping, Sweden.

**Corresponding authors: johanna.rosen@liu.se*

Table of Contents

Figure S 1: SQS information for single sheets. 3

Figure S 2: SQS convergence for single sheets. 3

Figure S 3: Comparison of binding energies calculated with Eq. 3. 4

Figure S 4: Topological energy surfaces for Cl, Br and I terminations adsorption 4

Figure S 5: Four termination adsorption sites for ss-MBene 5

Figure S 6: Topological energy surfaces for (1x1x1) ml-MBene cell. 5

Figure S 7: Phonon dispersion of ml-$Ti_2B_2T_x$ MBenes (T=Cl, Br and I). 6

Figure S 8: Phonon dispersion of ss-$Ti_2B_2T_x$ MBenes (T=Cl, Br and I). 6

Figure S 9: Charge difference comparison of ideal ml-$Ti_2B_2T_x$ MBenes (T=Cl, Br and I). 7

Figure S 10: Cl-terminated ml-MBenes relaxed structures from x=0 to x=2 (ideal coverage). 7

Figure S 11: Br-terminated ml-MBenes relaxed structures from x=0 to x=2 (ideal coverage). 8

Figure S 12: I-terminated ml-MBenes relaxed structures from x=0 to x=2 (ideal coverage). 8

Figure S 13: a-LP results for ss-MBenes 9

Figure S 14: DOS Comparison for different multilayer and single sheet MBenes. 10

Figure S 15: DOS for ml-MBenes with ideal termination coverage (x=0 or x=2 in $Ti_2B_2T_x$). 11

Figure S 16: DOS Evolution for Br-terminated ml-MBenes with halogen coverage. 11

Figure S 17: DOS Evolution for I-terminated ml-MBenes with halogen coverage. 12

Figure S 18: DOS Evolution for Cl-terminated ss-MBenes with halogen coverage. 12

Figure S 19: DOS Evolution for Br-terminated ss-MBenes with halogen coverage. 13

Figure S 20: DOS Evolution for I-terminated ss-MBenes with halogen coverage. 13

Figure S 21: COHP bonding and Bader charge analyses. 14

Figure S 22: PES for (1x1x1) ml-Ti_2CT_x MXenes, T_x=Br, Cl and I. 14

Figure S 23: Temperature evolution during ab initio molecular dynamics simulations. 15

Figure S 24: Evolution of termination-Ti distances during ab initio molecular dynamics. 15

Figure S 25: Evolution of I-Ti distances during ab initio molecular dynamics 16

Table S 1: Average Bader charges calculated for different $Ti_2B_2T_2$ ml/ss-MBenes. 17

Table S 2: Average Lowedin charges calculated for different $Ti_2B_2T_2$ ml/ss-MBenes. 17

References 18

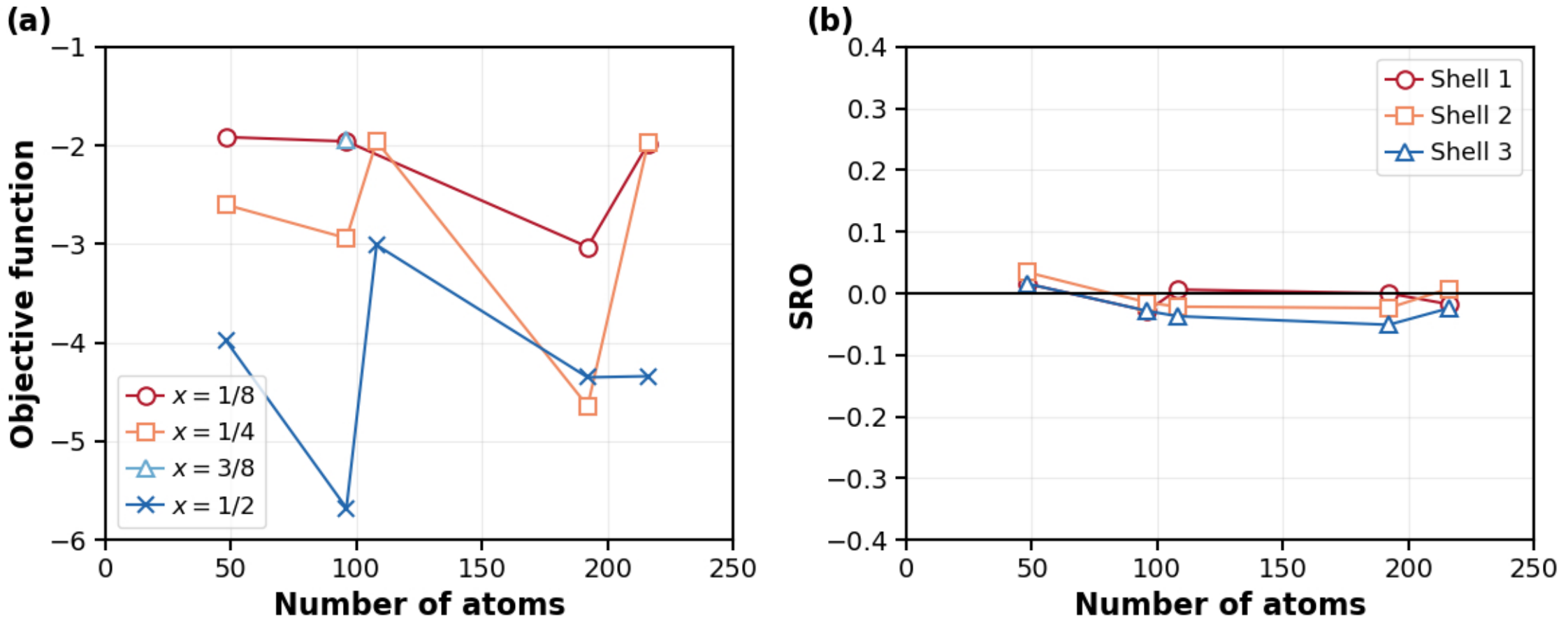

Figure S 1: SQS information for single sheets.

(a) Objective function of SQS supercells with different concentrations x. (b) SQS calculated Warren-Cowley short-range parameter (SRO) for different shells, according to ref [1]. Each SQS generation used a Monte Carlo algorithm for 1 day, with pair and triple cutoffs of 15 and 10 Å, respectively.

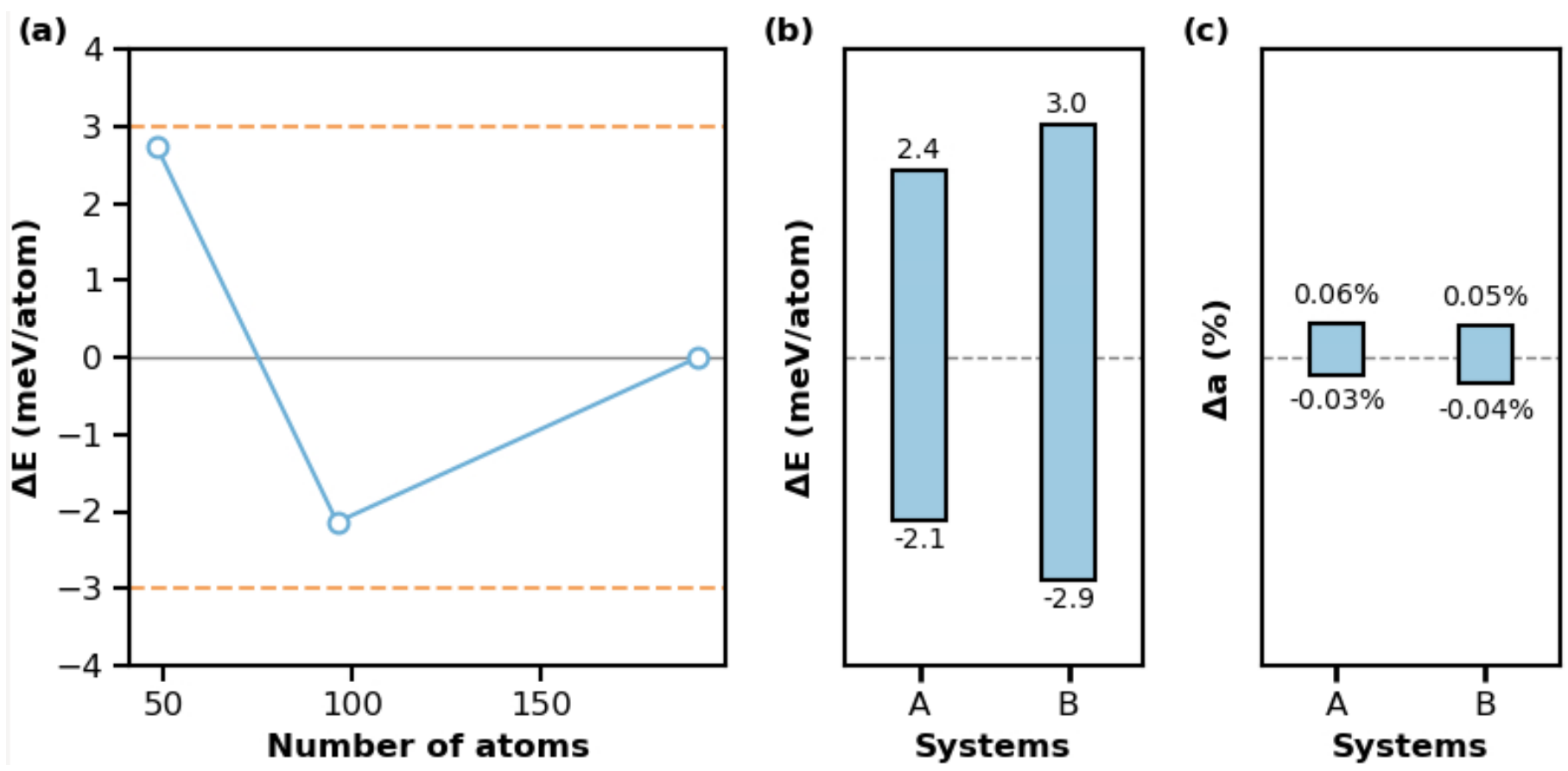

Figure S 2: SQS convergence for single sheets.

(a) Energy difference (ΔE) with respect to the largest supercell of $Ti_2B_2Cl_{0.25}O_{1.75}$ material. The dashed lines represent the ± 3 meV/atom convergence criteria used here. It was estimated by calculating the energy and a-lattice parameter differences for three SQS cells, as shown in (b) and (c). Systems A and B refer to, respectively, $Ti_2B_2Br_{1.5}O_{1.5}$ and $Ti_2B_2Cl_{0.5}$ compositions.

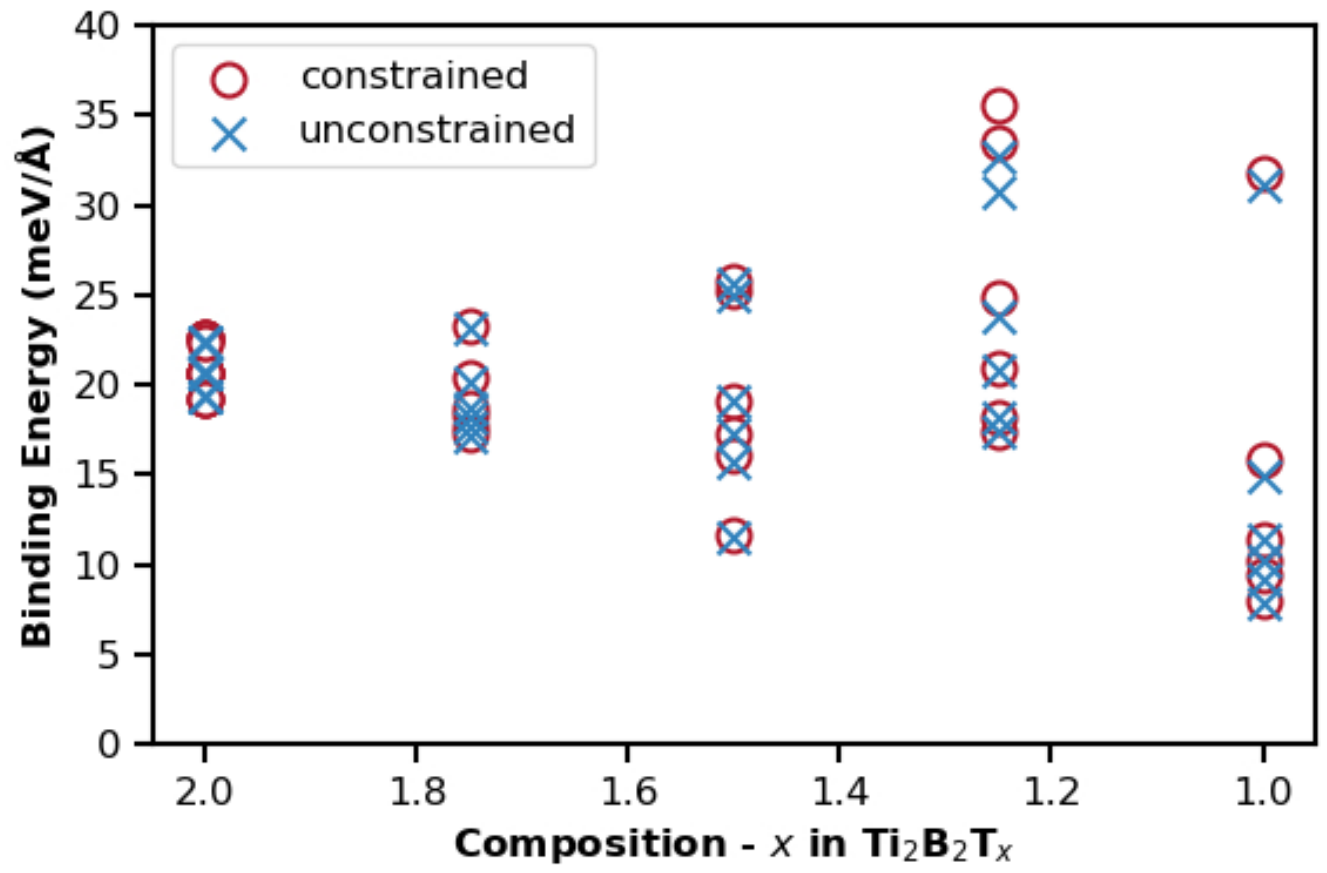

Figure S 3: Comparison of binding energies calculated with Eq. 3.

Constrained refers to calculations relaxing only atomic positions while unconstrained also enabled lattice-parameter relaxation. Note that the numbers are very similar because of the similar ml-and ss- MBenes surface areas.

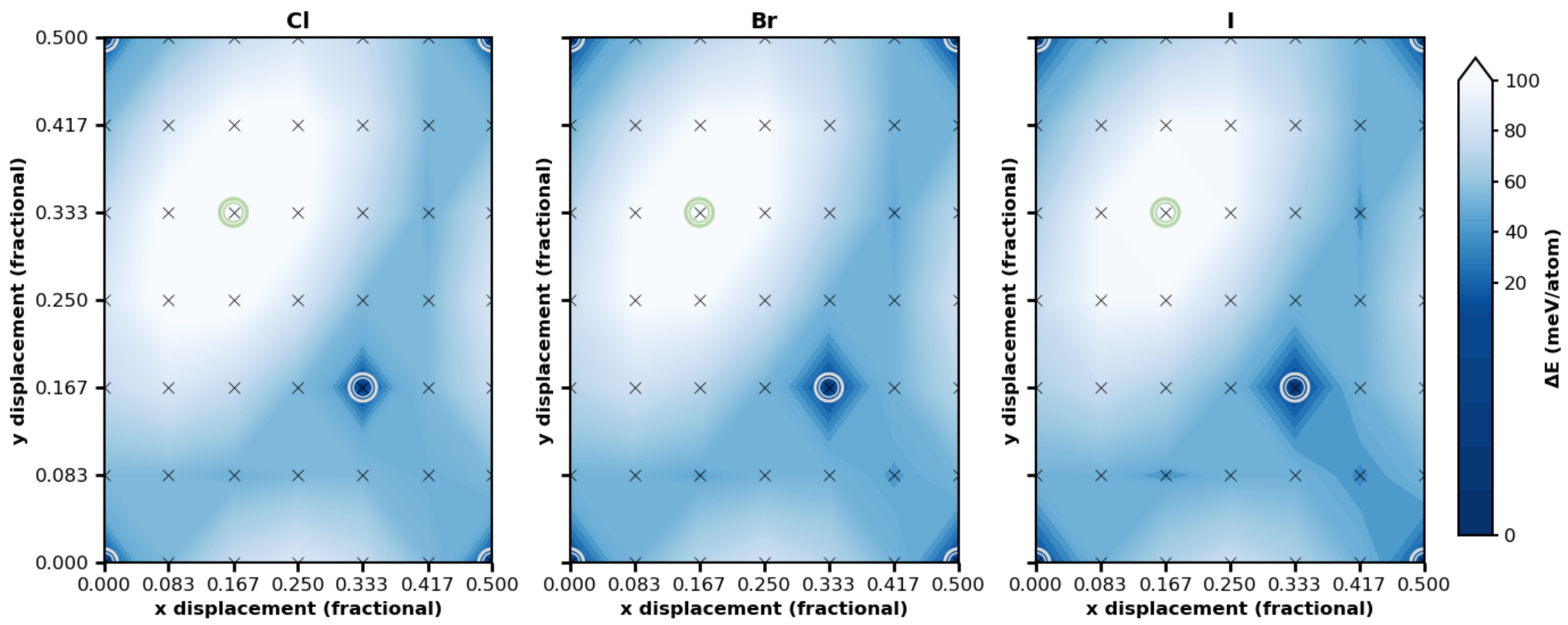

Figure S 4: Topological energy surfaces for Cl, Br and I terminations adsorption

These calculations considered only two terminations per (2x2x1) ss-MBene supercell. All atoms were kept in the x and y- atomic positions, while they were allowed to move freely in the z-direction (the volume was also relaxed). The colorbar is not linear to better represent the most stable terminations sites. The green and grey circles represent, respectively, Ti and B atoms sites.

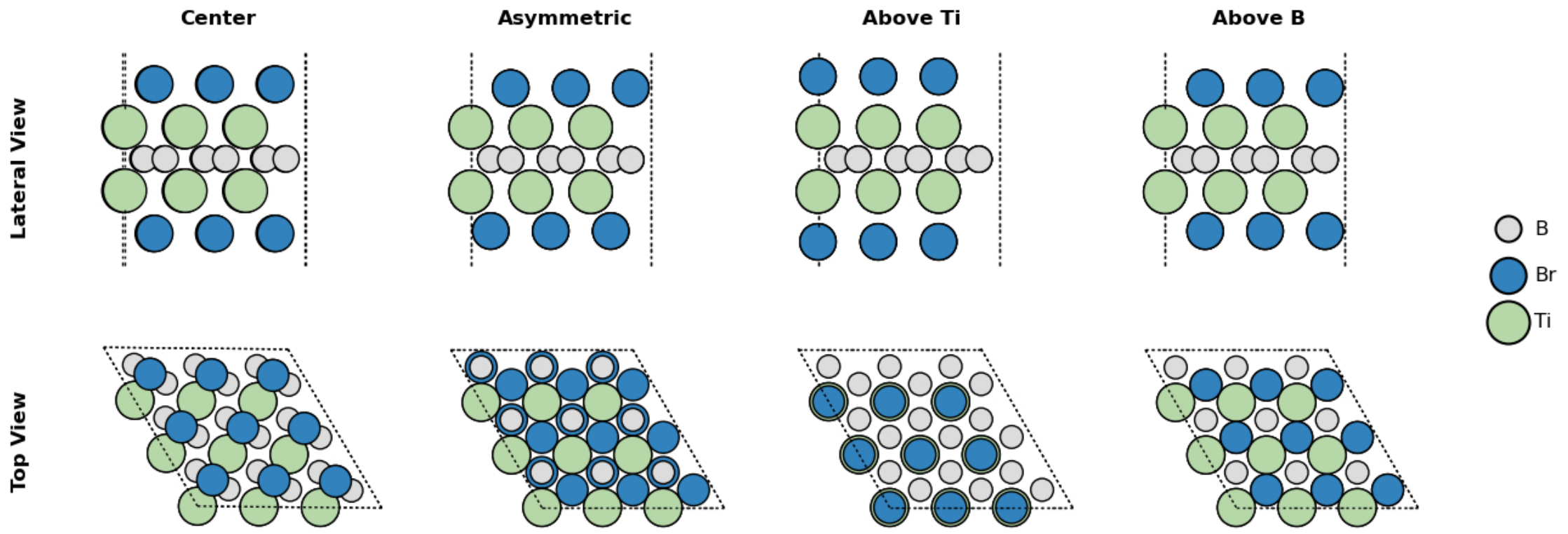

Figure S 5: Four termination adsorption sites for ss-MBene

The cell was allowed to relax all atomic positions, volume and shape. The most energetically favourable one was 'Above B'.

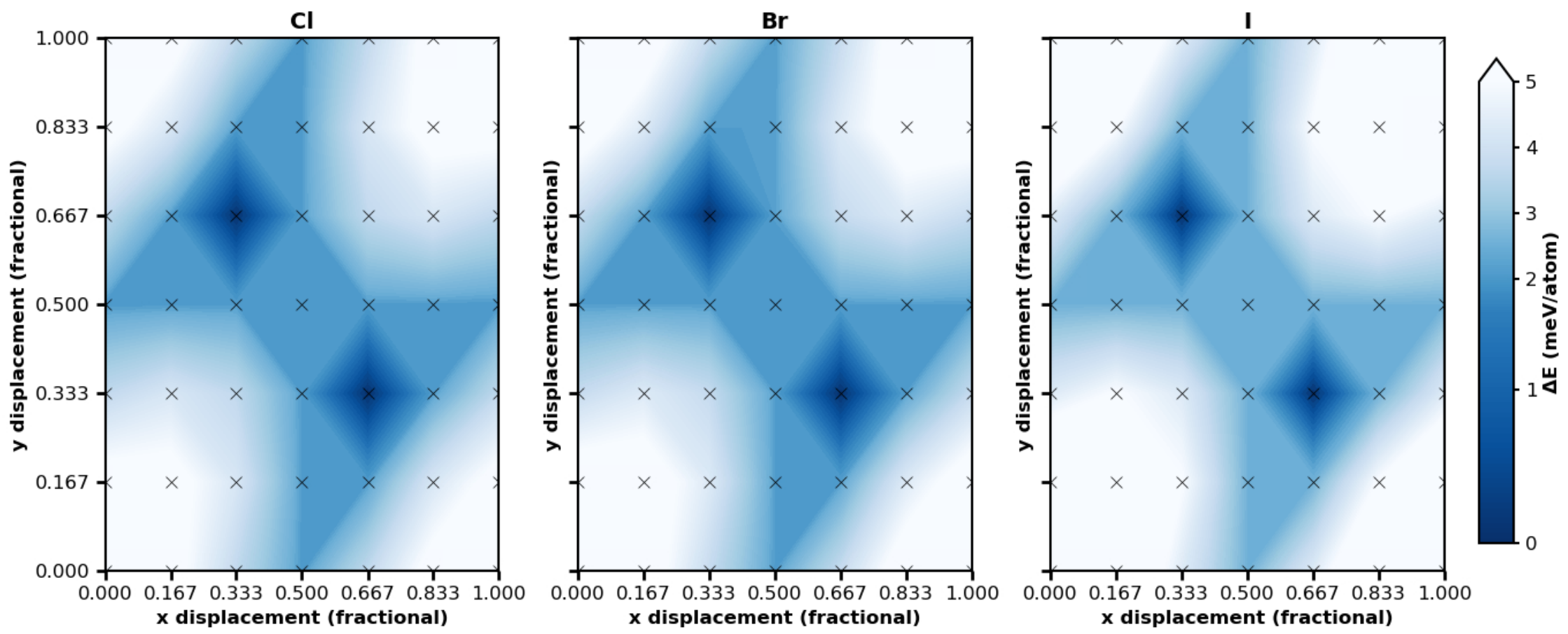

Figure S 6: Topological energy surfaces for (1x1x1) ml-MBene cell.

All atoms were kept fixed in x and y coordinates, while being allowed to move freely in the z-direction only (the volume was also relaxed). The colorbar is not linear to better represent the most stable stacking configurations.

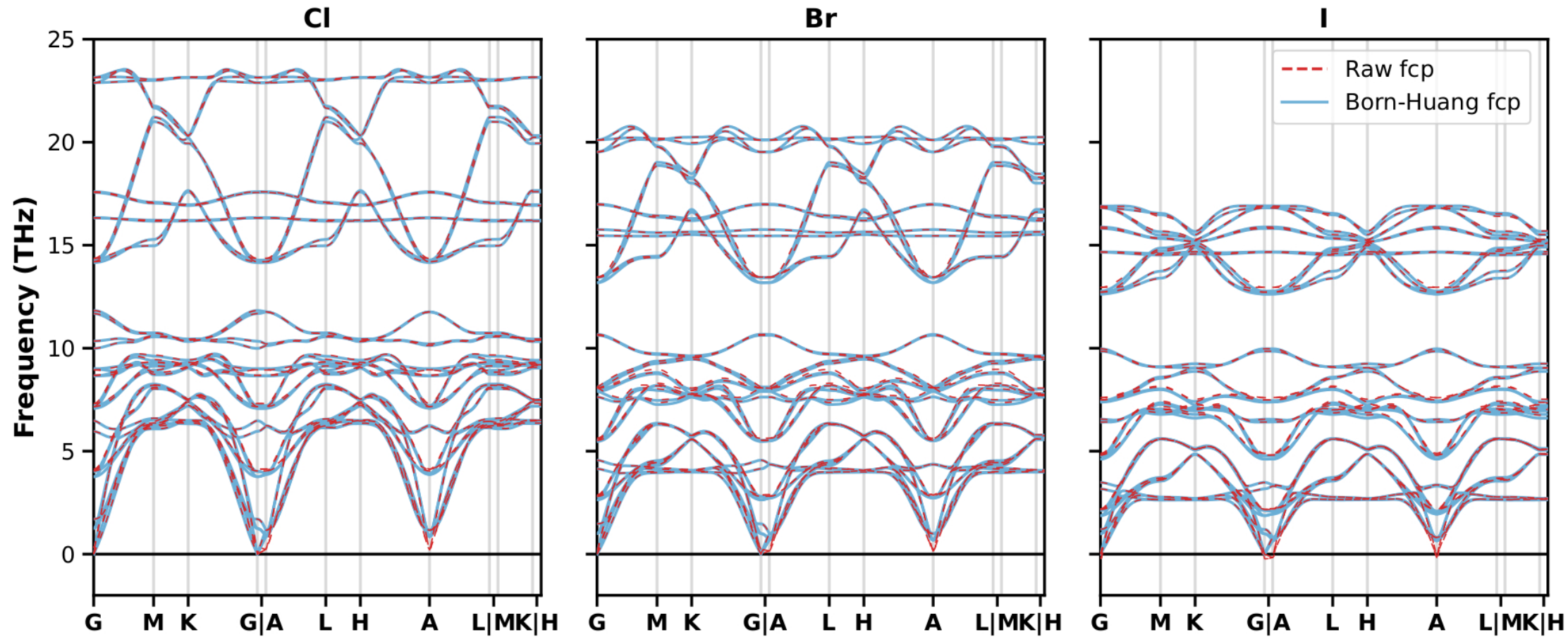

Figure S 7: Phonon dispersion of ml-$Ti_2B_2T_x$ MBenes (T=Cl, Br and I).

The dashed red lines and solid blue ones represent the uncorrected and Born-Huang constrained force constants, respectively. The high symmetry path was obtained following ref [2].

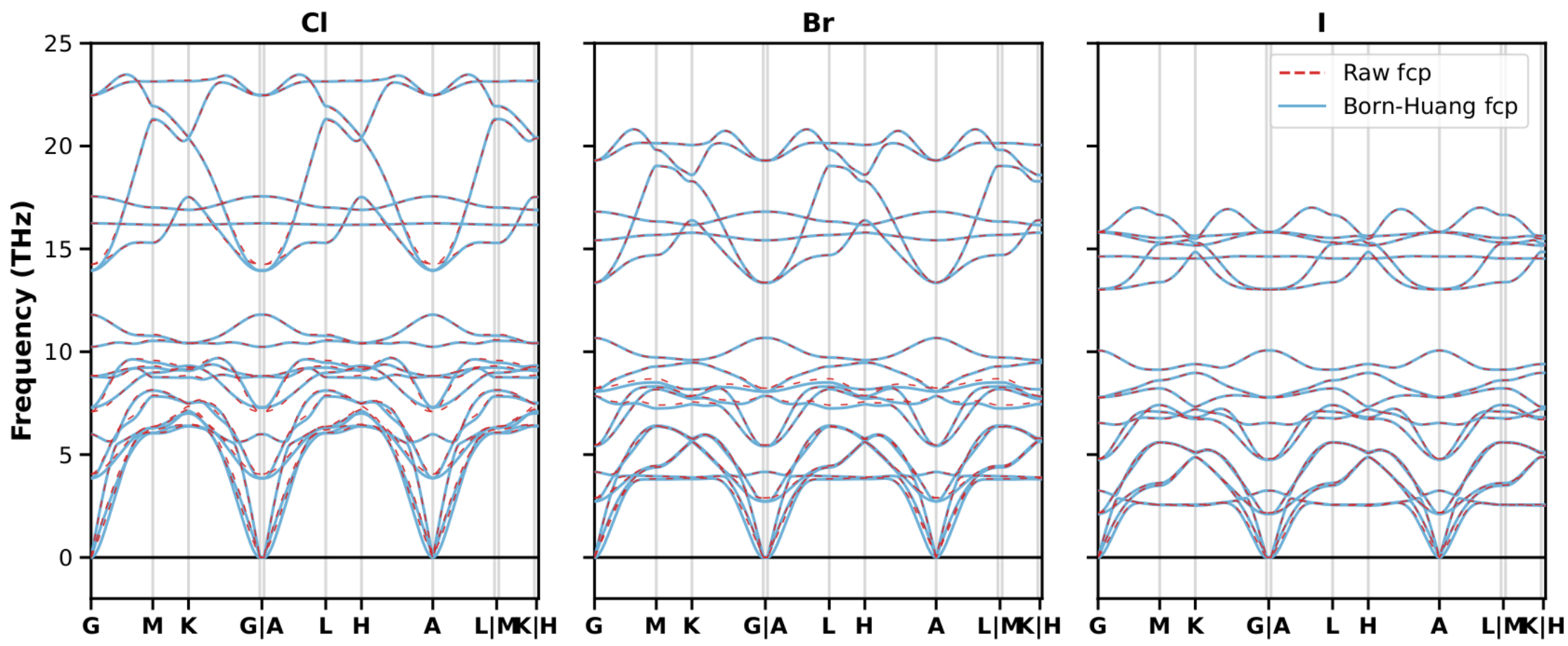

Figure S 8: Phonon dispersion of ss-$Ti_2B_2T_x$ MBenes (T=Cl, Br and I).

The dashed red lines and solid blue ones represent the uncorrected and Born-Huang constrained force constants, respectively. The high symmetry path was obtained following ref [2].

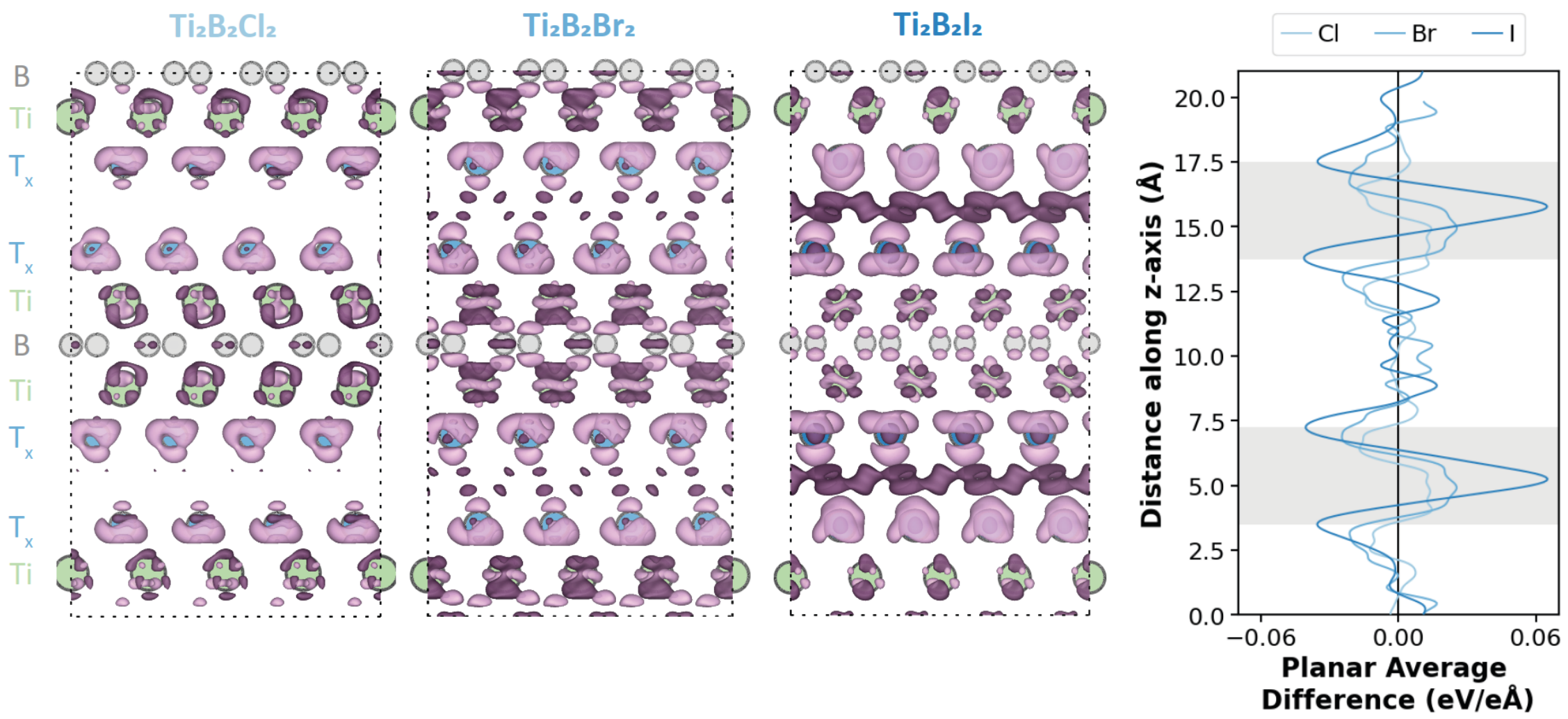

Figure S 9: Charge difference comparison of ideal ml-$Ti_2B_2T_x$ MBenes (T=Cl, Br and I).

The charge difference was calculated as the difference between ml-structure and the individual layers present on them, i.e., $\Delta\rho = \rho_{ml} - (\rho_{layer\ 1} + \rho_{layer\ 2})$. Isosurfaces values were kept constant (1E^{-4} e/A^3), while the dark (light) pink regions represent electron density accumulation (depletion). The planar-average charge density difference was computed along the z-axis direction, with the interlayer region highlighted in gray.

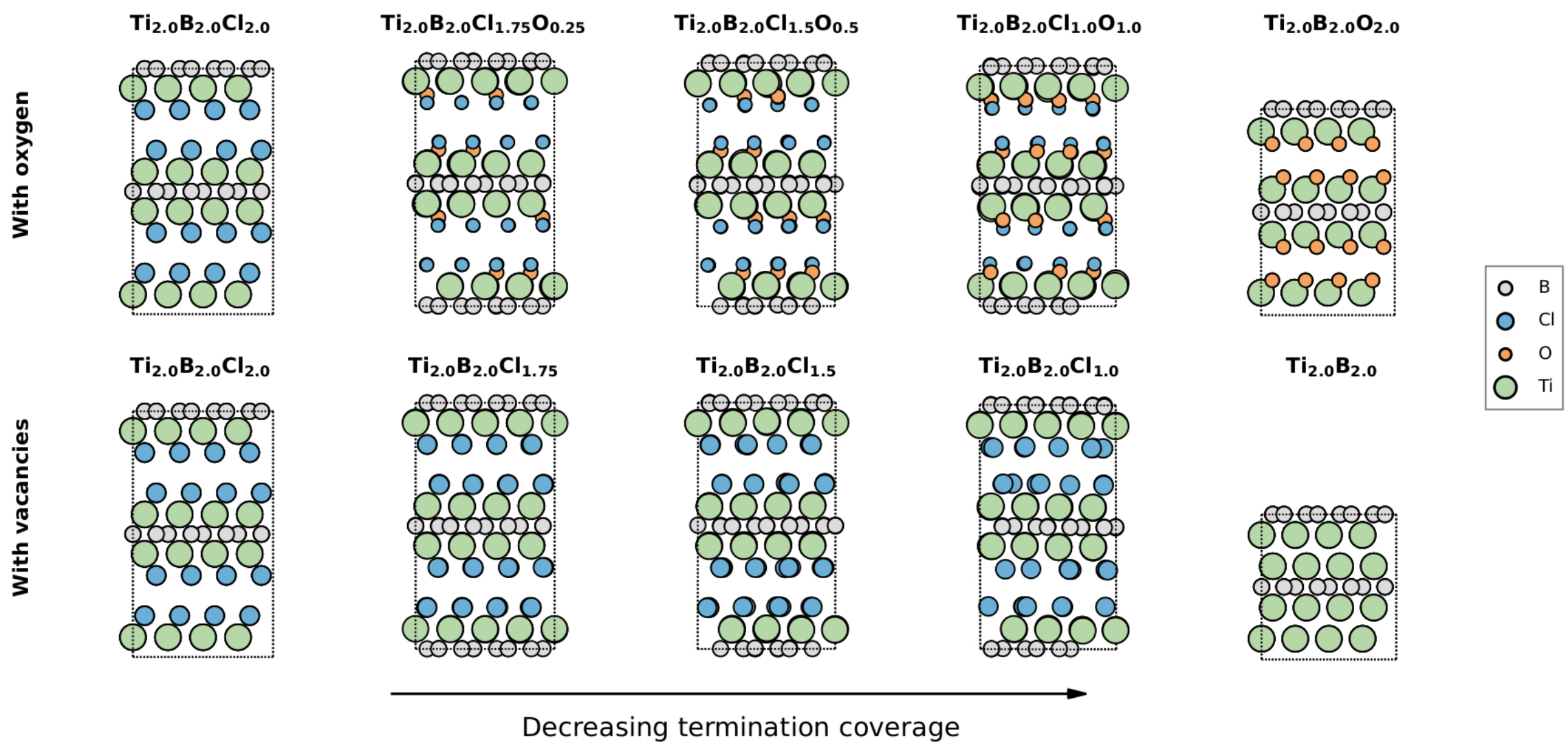

Figure S 10: Relaxed structures of ml-MBenes with varying Cl coverage. Relaxed structures of ml-MBenes with Cl coverage ranging from x = 0 to x = 2, corresponding to no Cl termination and full Cl termination, respectively. From x = 2 to x = 0, the removed Cl terminations are replaced either by O terminations (top) or by vacant termination sites (bottom).

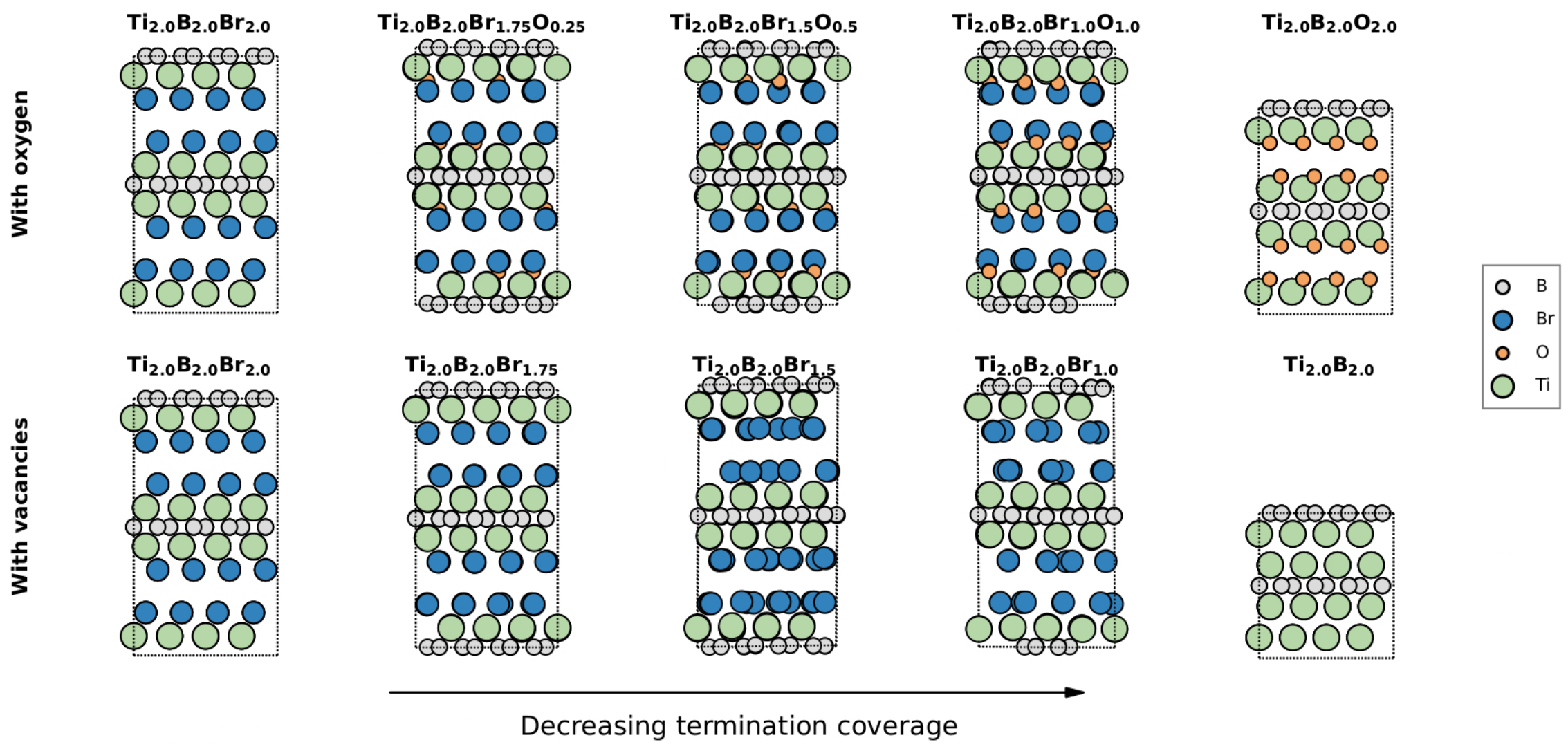

Figure S 11: Relaxed structures of ml-MBenes with varying Br coverage. Relaxed structures of ml-MBenes with Br coverage ranging from x = 0 to x = 2, corresponding to no Br termination and full Br termination, respectively. From x = 2 to x = 0, the removed Br terminations are replaced either by O terminations (top) or by vacant termination sites (bottom).

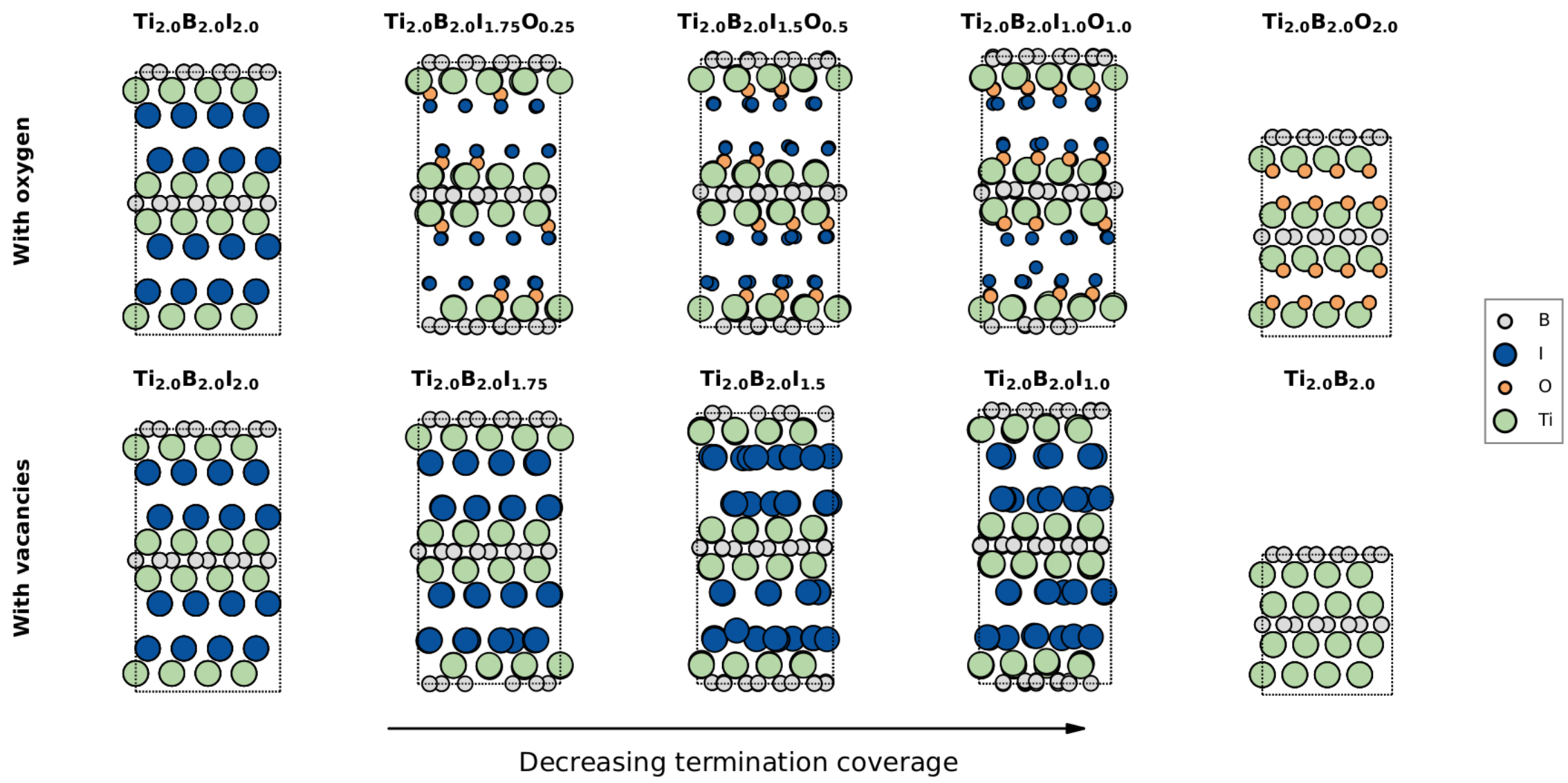

Figure S 12: Relaxed structures of ml-MBenes with varying I coverage. Relaxed structures of ml-MBenes with I coverage ranging from x = 0 to x = 2, corresponding to no I termination and full I termination, respectively. From x = 2 to x = 0, the removed I terminations are replaced either by O terminations (top) or by vacant termination sites (bottom).

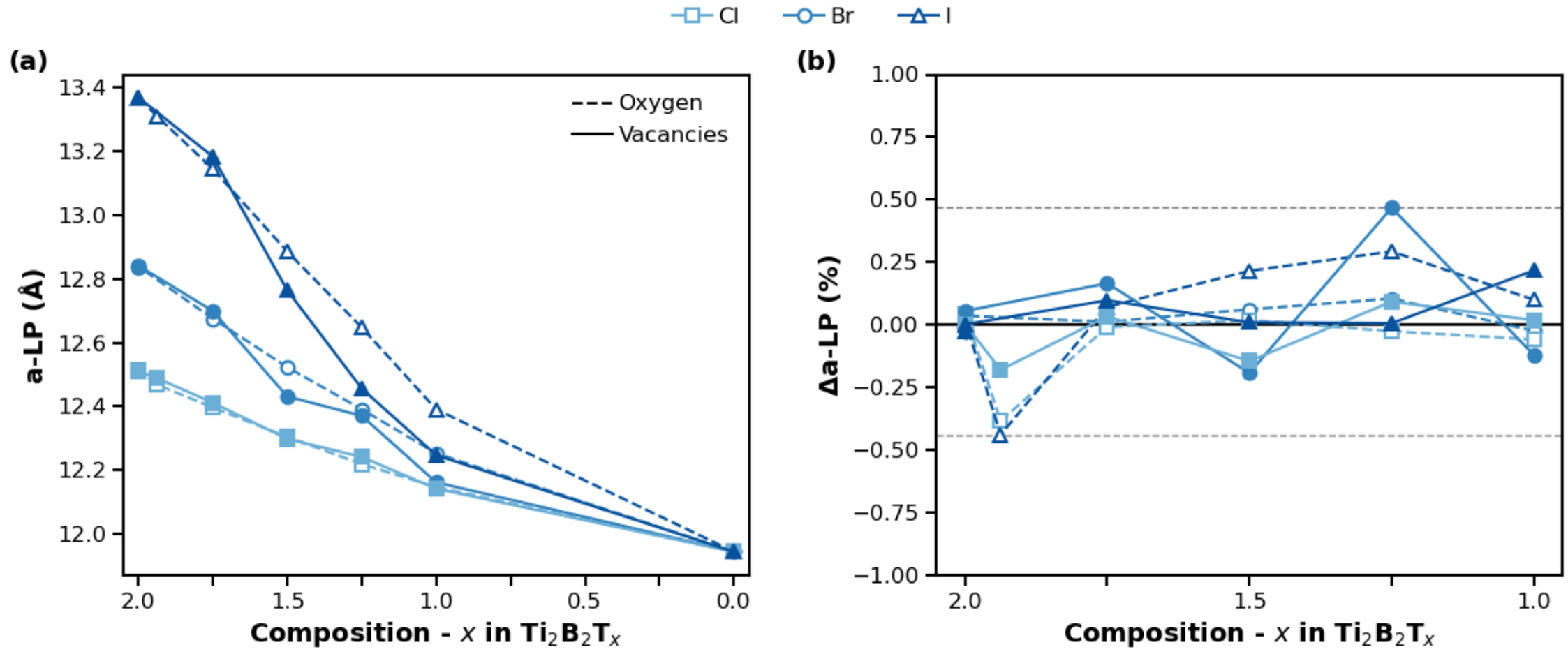

Figure S 13: a-LP results for ss-MBenes

(a) Relaxed a-lattice parameters for ss-MBenes (b) Relative difference of relaxed a-LP parameters for ss- and ml- structures.

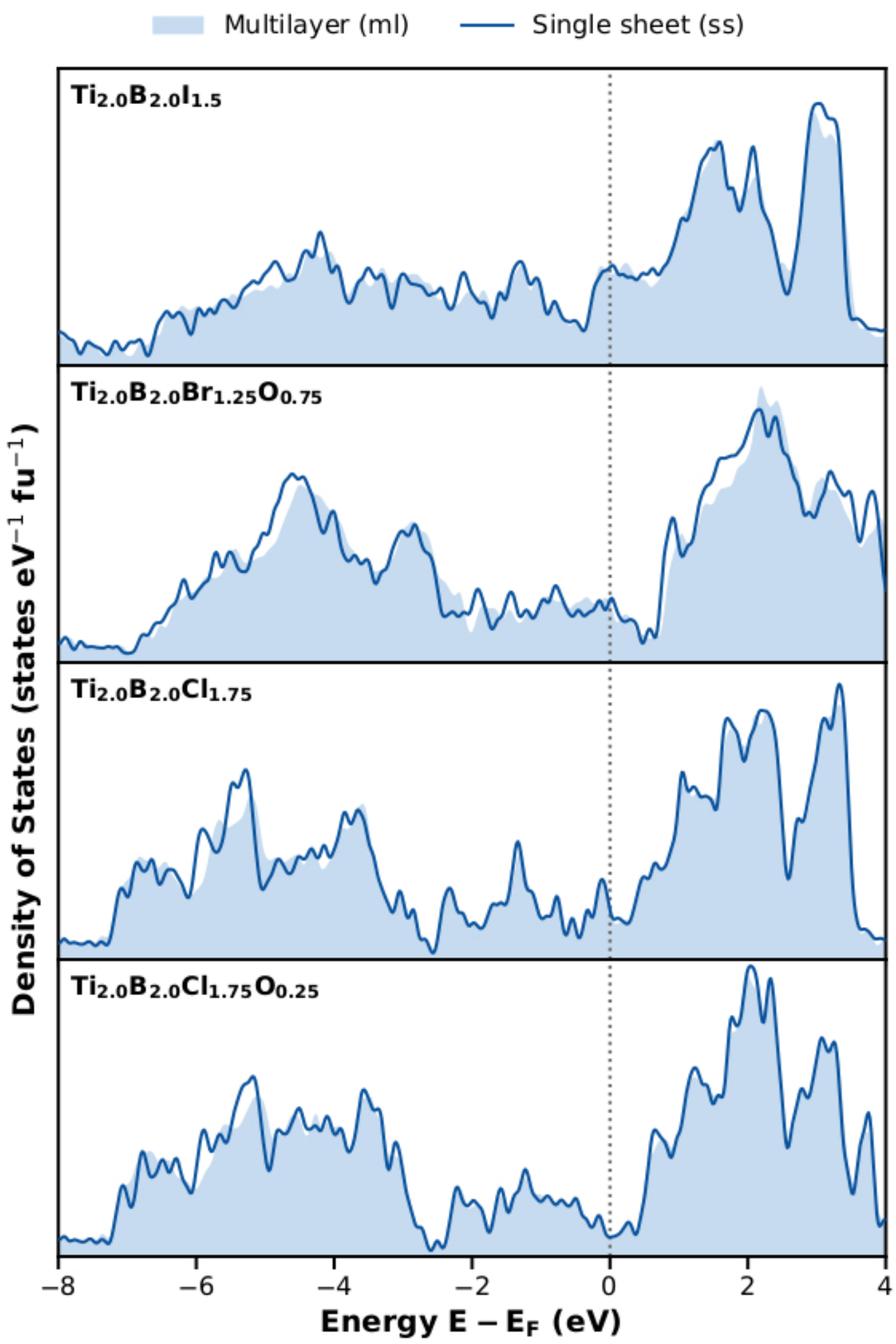

Figure S 14: DOS Comparison for different multilayer and single sheet MBenes.

The chosen materials encompass different terminations atoms as well as the presence or not of oxygen. Note that the DOS per f.u. between ml and ss are almost identical for all cases.

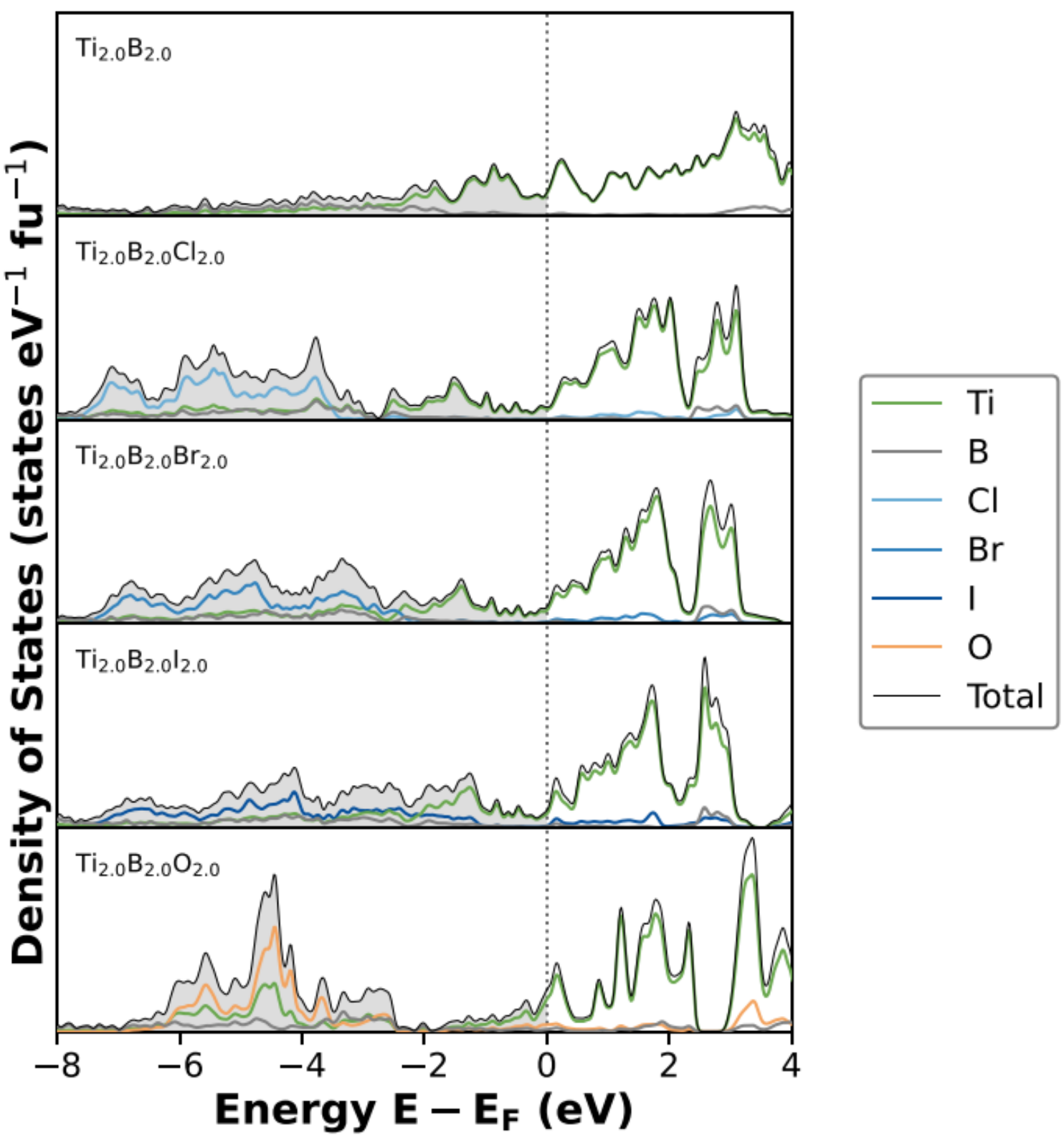

Figure S 15: DOS for ml-MBenes with ideal termination coverage (x=0 or x=2 in $Ti_2B_2T_x$). The density of states (y-axis) is normalized per formula unit (f.u.) and the shaded regions schematically denote the occupied (filled) electronic states up to the Fermi level (E_F).

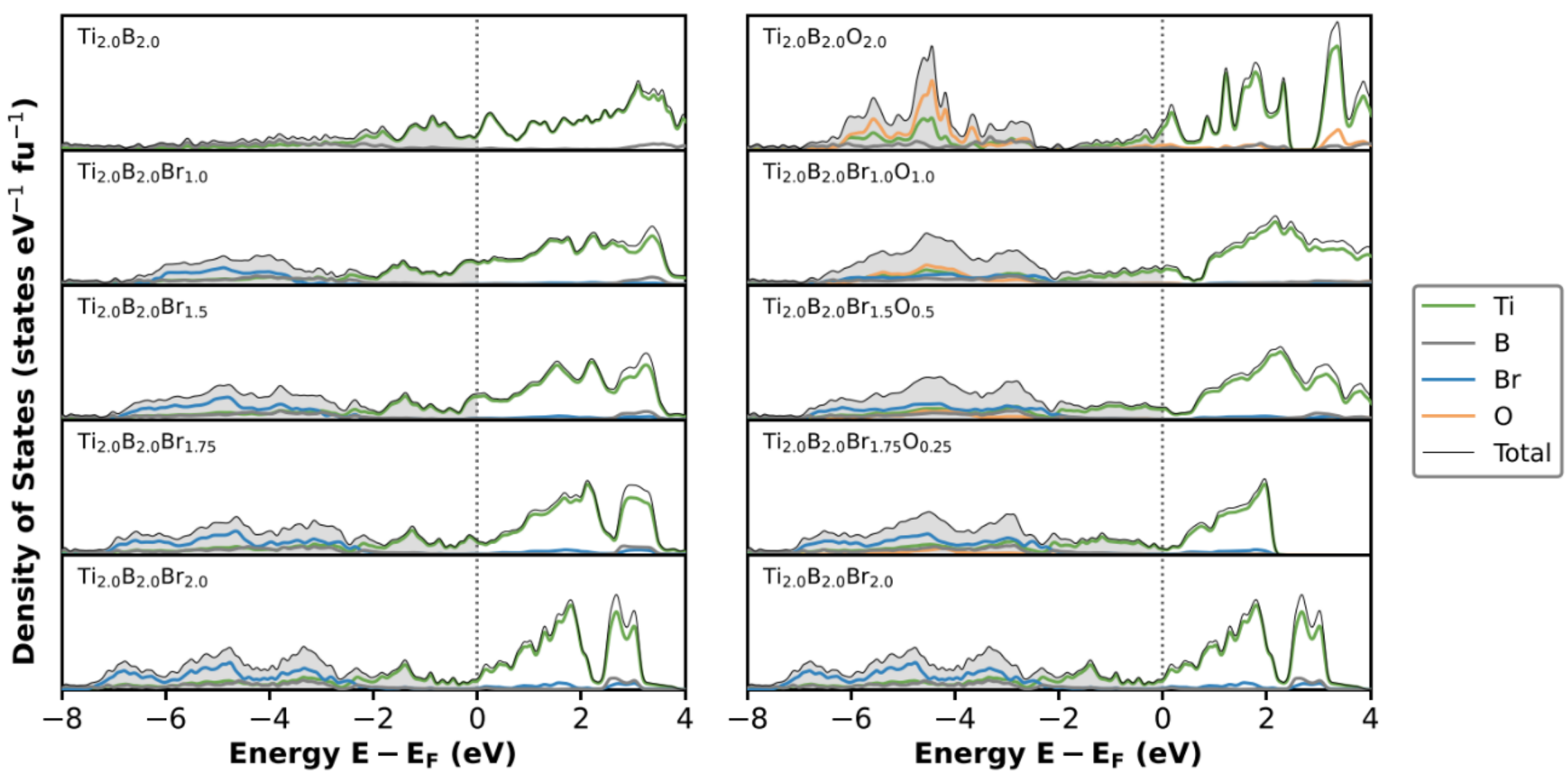

Figure S 16: DOS Evolution for Br-terminated ml-MBenes with halogen coverage. The density of states (y-axis) is normalized per formula unit (f.u.) and the shaded regions schematically denote the occupied (filled) electronic states up to the Fermi level (E_F).

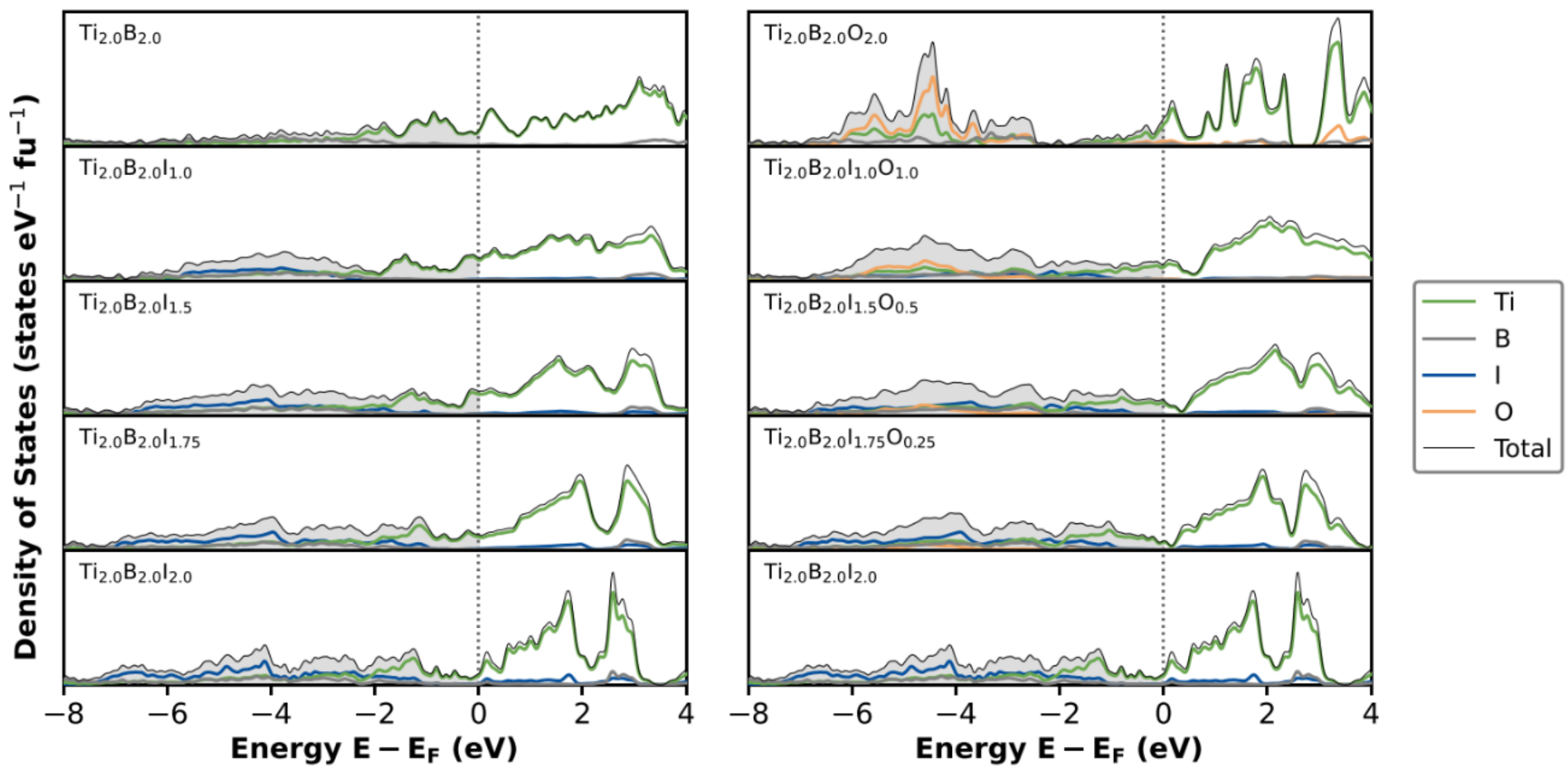

Figure S 17: DOS Evolution for I-terminated mI-MBenes with halogen coverage.
The density of states (y-axis) is normalized per formula unit (f.u.) and the shaded regions schematically denote the occupied (filled) electronic states up to the Fermi level (E_F).

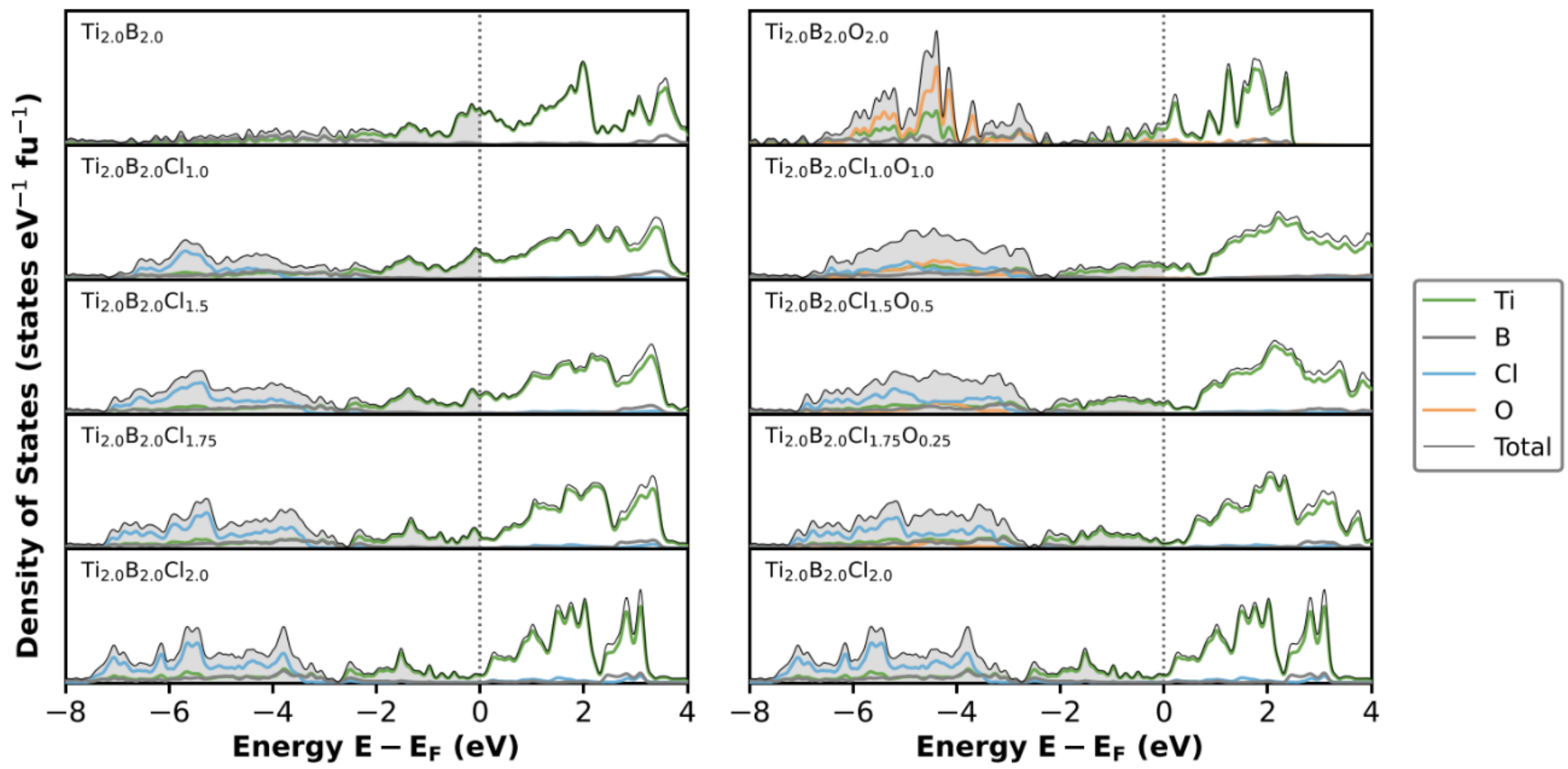

Figure S 18: DOS Evolution for Cl-terminated ss-MBenes with halogen coverage.
The density of states (y-axis) is normalized per formula unit (f.u.) and the shaded regions schematically denote the occupied (filled) electronic states up to the Fermi level (E_F).

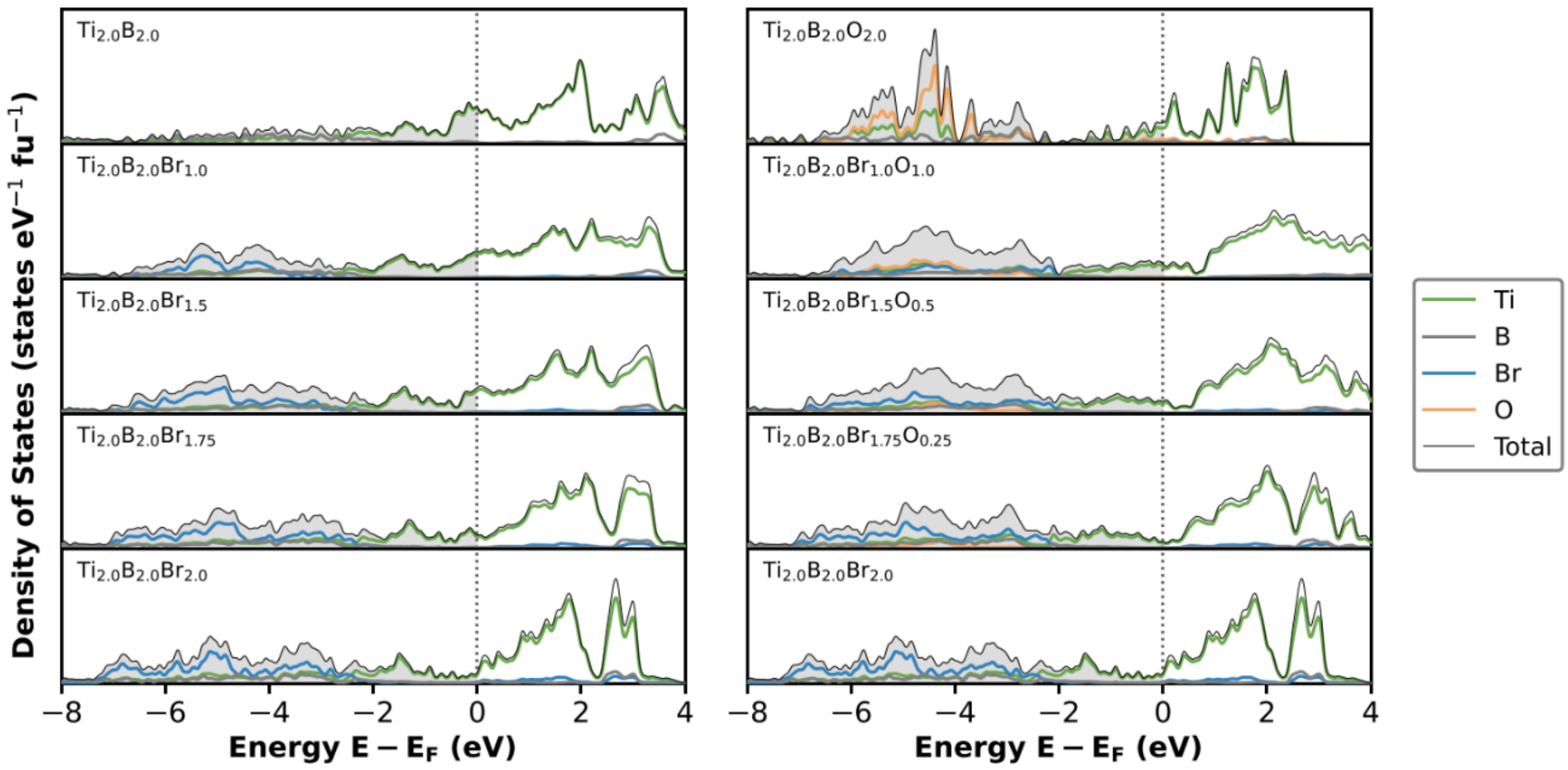

Figure S 19: DOS Evolution for Br-terminated ss-MBenes with halogen coverage. The density of states (y-axis) is normalized per formula unit (f.u.) and the shaded regions schematically denote the occupied (filled) electronic states up to the Fermi level (E_F).

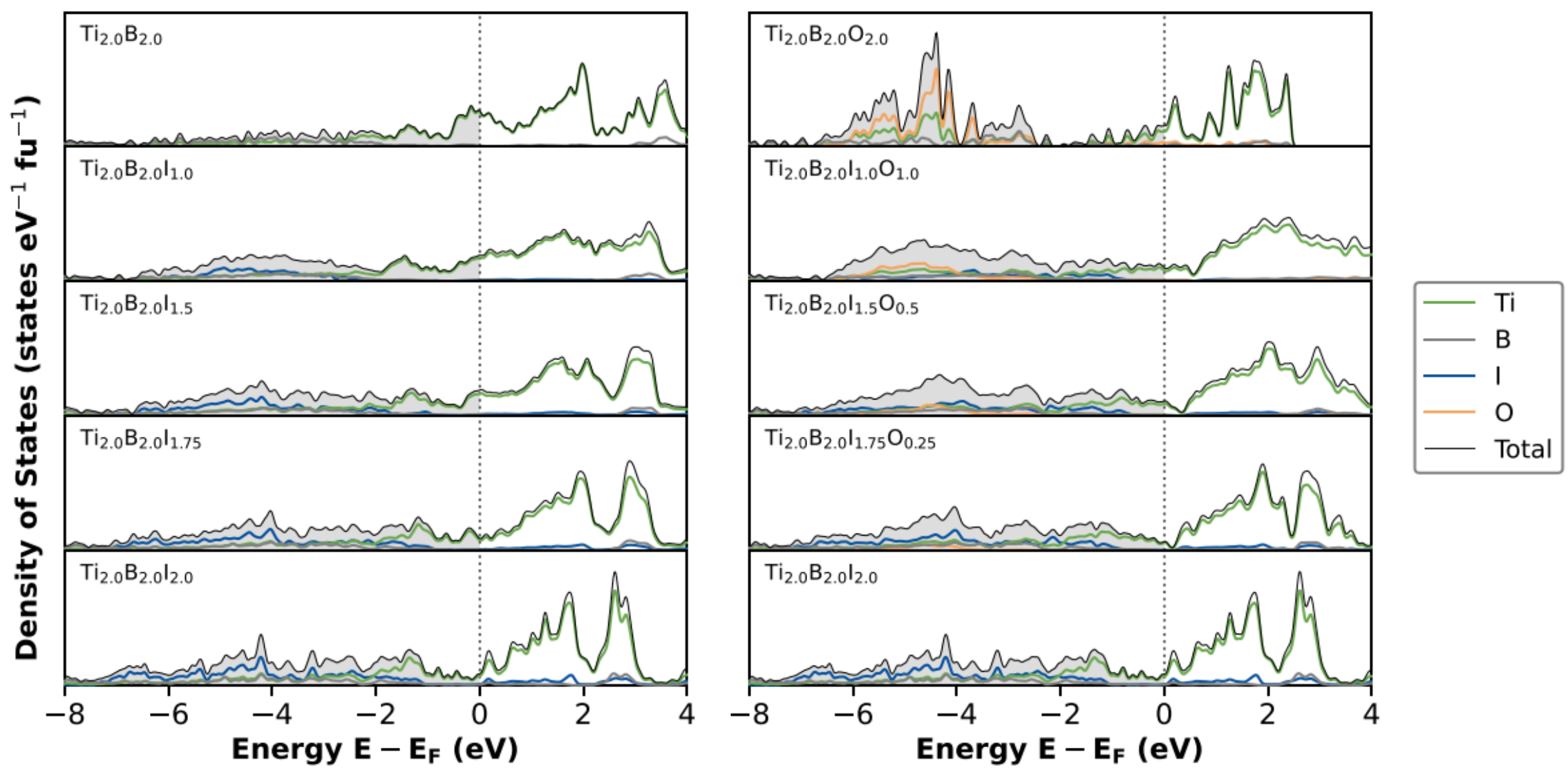

Figure S 20: DOS Evolution for I-terminated ss-MBenes with halogen coverage. The density of states (y-axis) is normalized per formula unit (f.u.) and the shaded regions schematically denote the occupied (filled) electronic states up to the Fermi level (E_F).

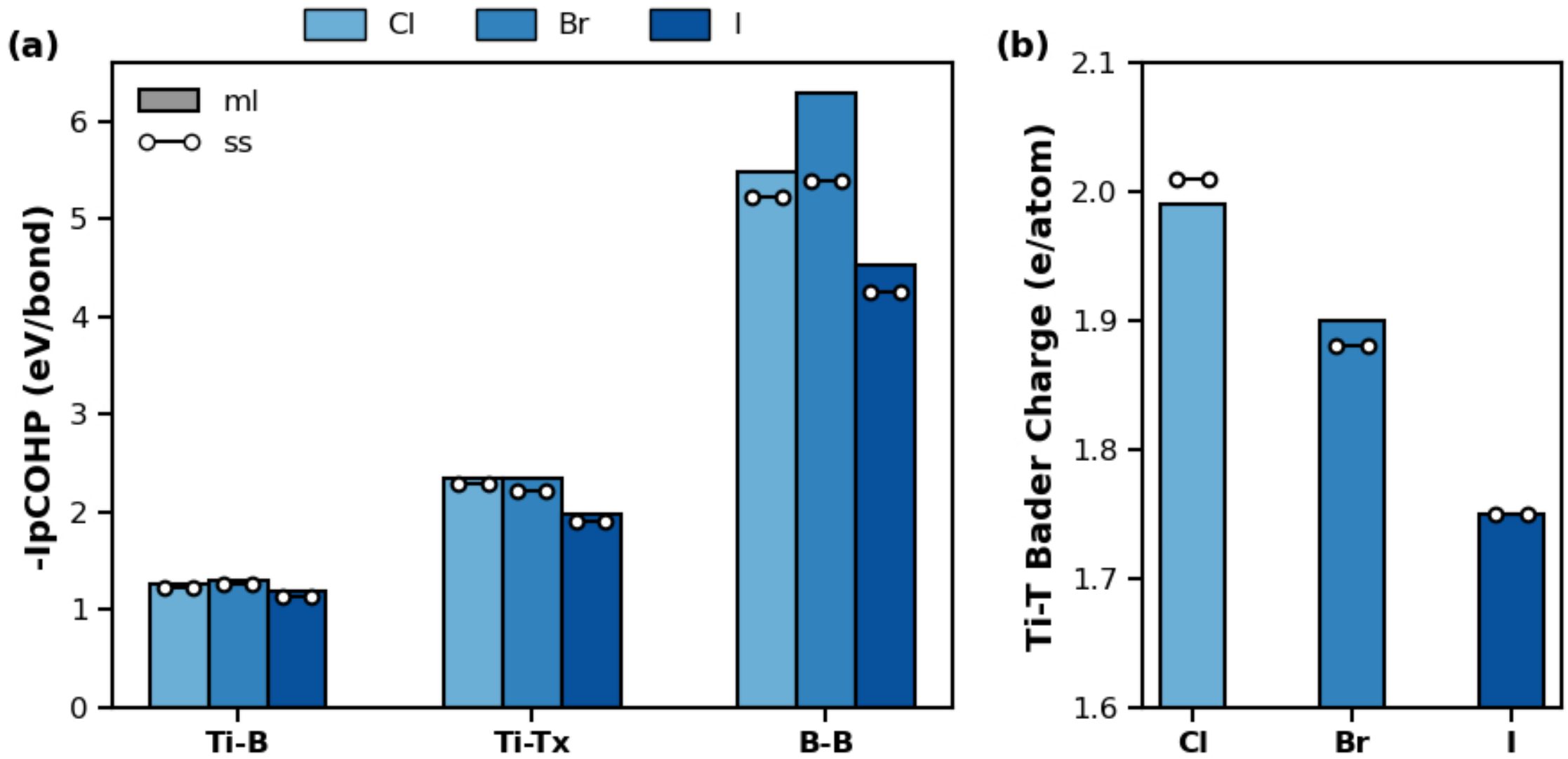

Figure S 21: COHP bonding and Bader charge analyses.

(a) Integrated -pCOHP occupied states averaged by the number of bonds for the first neighbor interactions. (b) Bader charge difference of Ti-T interactions. The bars represent the ml-values, while the lines show their respective values in the ss-$Ti_2B_2T_x$ MBenes. Complete data values are shown in Tables Table S 1Table S 2.

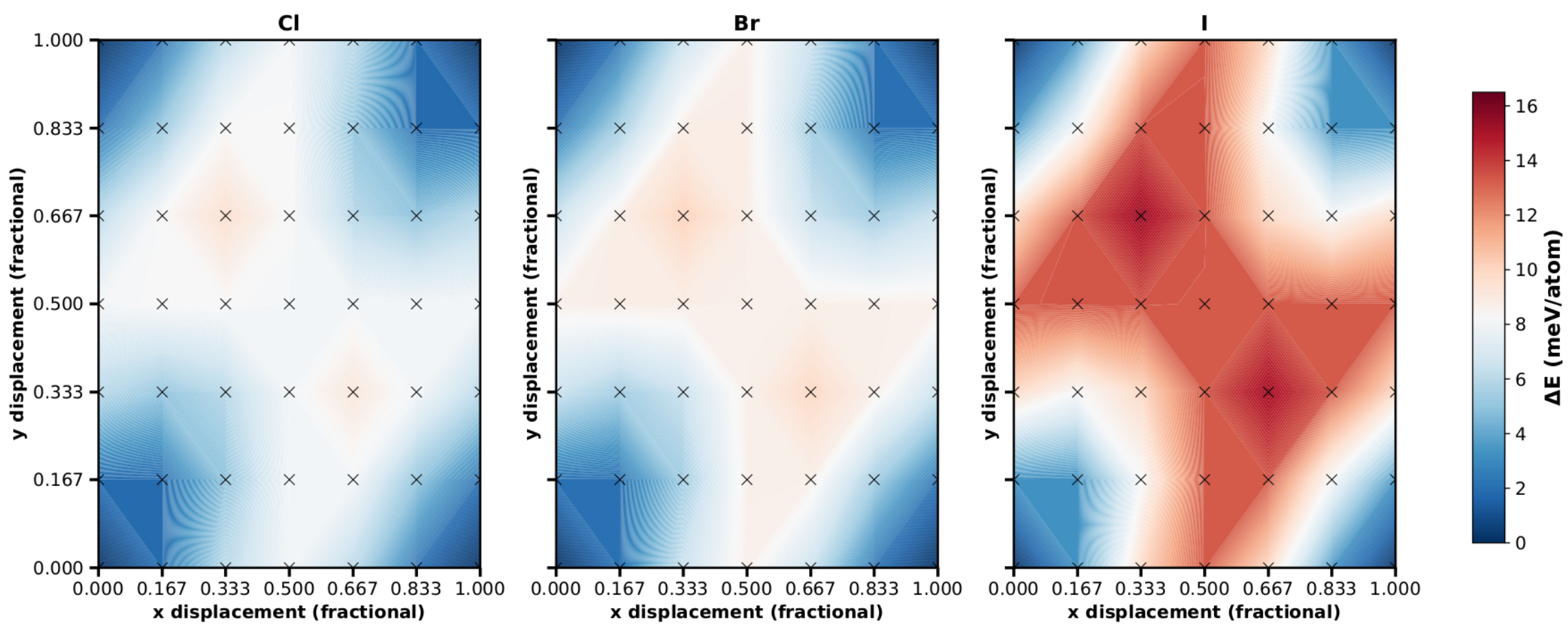

Figure S 22: PES for (1x1x1) ml-Ti_2CT_x MXenes, T_x=Br, Cl and I.

All atomic positions, cell shape and volume were kept fixed (single point calculations). The colorbar range is the same as Figure 5, to make the comparison easier.

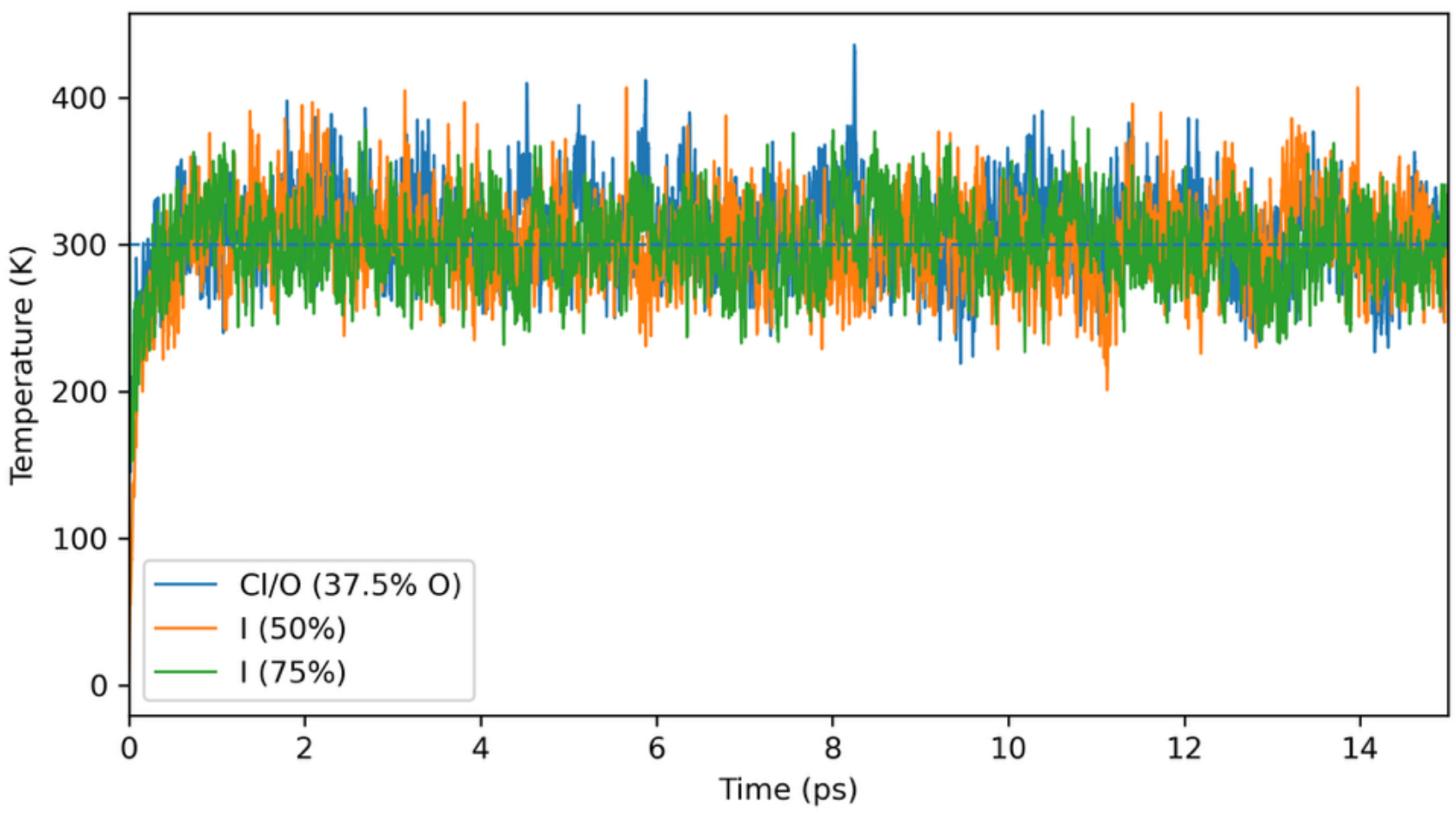

Figure S 23: Temperature evolution during ab initio molecular dynamics simulations.

Temperature evolution during NVT ab initio molecular dynamics simulations of representative termination configurations at a target temperature of 300 K. The simulations were performed with a 1 fs timestep using the Langevin thermostat with a friction coefficient of 10 ps^{-1} for all elements. The three trajectories correspond to the Cl/O-terminated MBene with 37.5% O substitution and the I-terminated MXenes with 50% and 75% I coverage, as indicated.

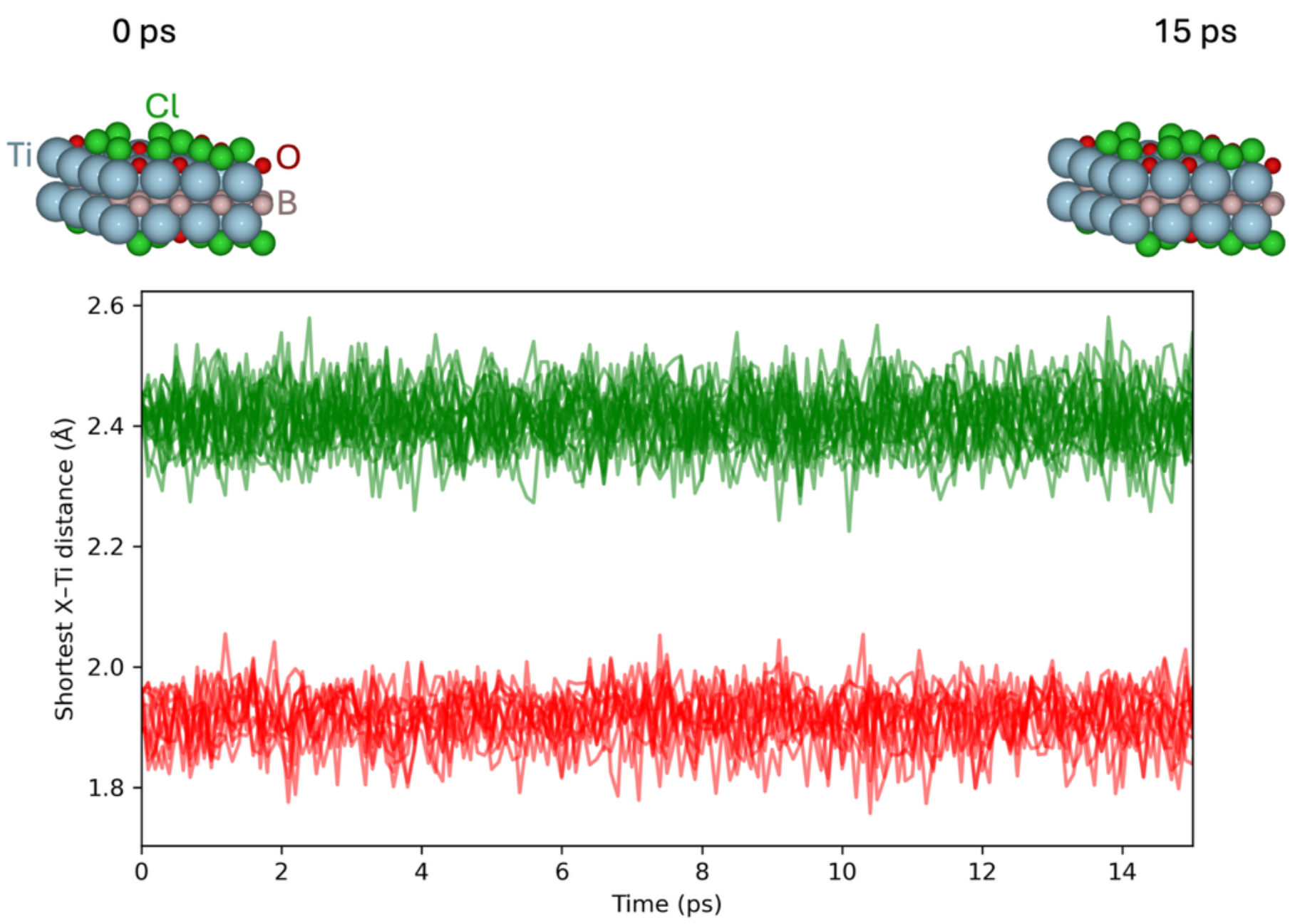

Figure S 24: Evolution of termination-Ti distances during ab initio molecular dynamics

Shortest Ti–O and Ti–Cl distances for each O and Cl termination, respectively, during the 300 K NVT ab initio molecular dynamics simulation of the Cl/O-terminated MXene with 37.5% O substitution. Red traces correspond to O–Ti distances and green traces to Cl–Ti distances. Each trace represents an individual termination site, with the distances calculated to the nearest Ti atom in each trajectory frame. Structural representations of the initial and final configurations are shown above the graph.

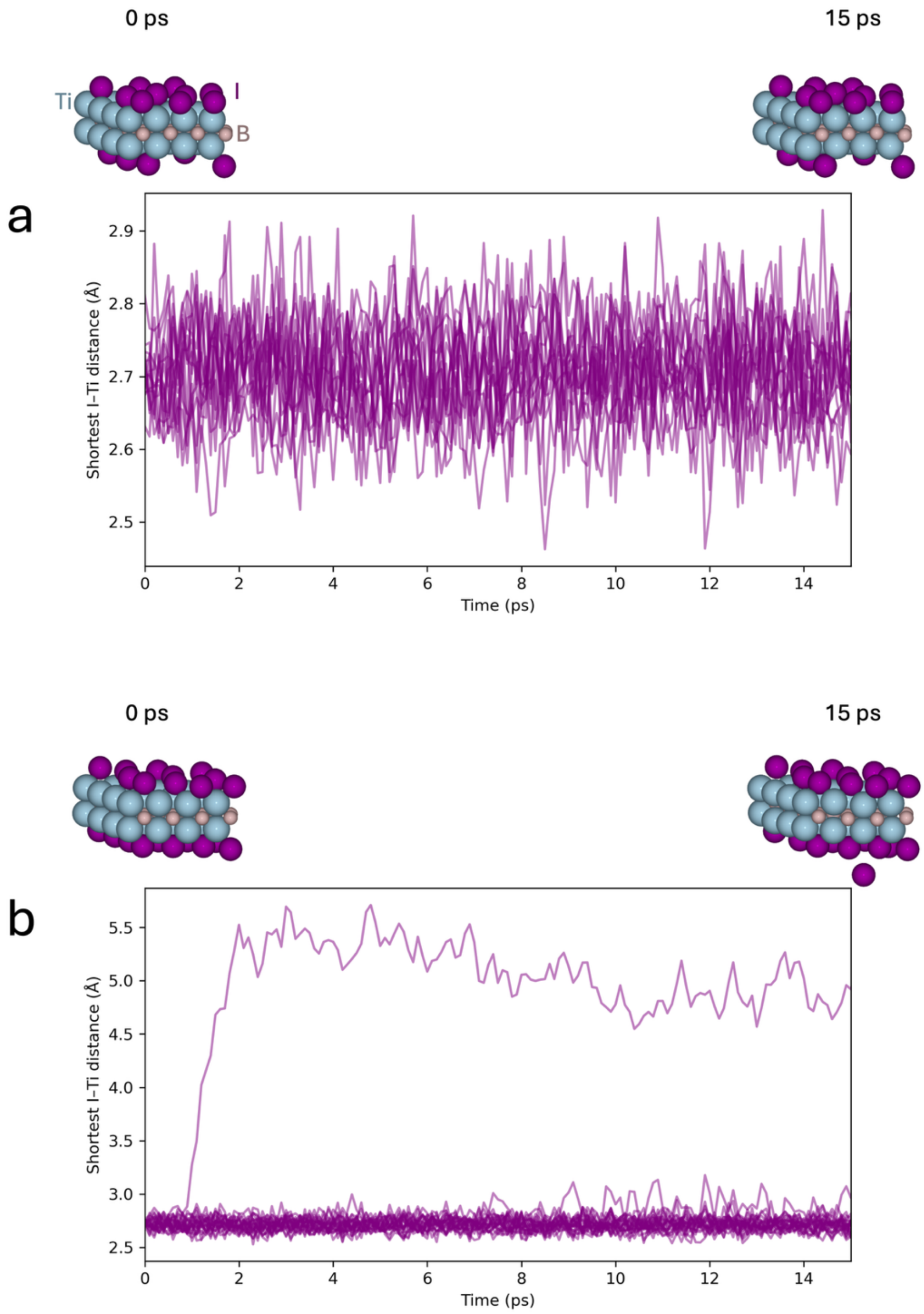

Figure S 25: Evolution of I-Ti distances during ab initio molecular dynamics

Shortest I–Ti distances for each I termination during 300 K NVT ab initio molecular dynamics simulations of the I-terminated MXenes with (a) 50% and (b) 75% I coverage. Each trace represents an individual I atom, with the distance calculated to the nearest Ti atom in each trajectory frame. Structural representations of the initial and final configurations are shown above respective graph. Notably, at 50% I coverage, the material retains its structural integrity, whereas at 75% I coverage, some I atoms diverge considerably from the MBene backbone.

	T = Cl	T = Br	T = I
Ti	1.39 / 1.39<br>[0.00]	1.35/ 1.39<br>[0.04]	1.28/ 1.28<br>[0.00]
B1	-0.86 / -0.87<br>[-0.01]	-0.89/ -0.89<br>[0.00]	-0.81/ -0.89<br>[0.08]
B2	-0.70 / -0.70<br>[0.00]	-0.71/ -0.72<br>[0.01]	-0.82/ -0.74<br>[-0.12]
T	-0.60 / -0.61<br>[0.01]	-0.55/ -0.54<br>[-0.01]	-0.47/ -0.47<br>[0.00]
Ti – T	1.99 / 2.01<br>[-0.02]	1.90 / 1.88<br>[0.02]	1.75 / 1.75<br>[0.00]

Table S 1: Average Bader charges calculated for different $Ti_2B_2T_2$ ml/ss-MBenes.

The difference between ml- and ss- values is showed inside squared brackets.

	T = Cl	T = Br	T = I
Ti	0.81 / 0.77<br>[-0.04]	0.83 / 0.79<br>[-0.04]	0.67 / 0.63<br>[-0.04]
B1	-0.58 / -0.53<br>[0.05]	-0.58 / -0.59<br>[-0.01]	-0.64 / -0.58<br>[0.06]
B2	-0.61 / -0.58<br>[0.03]	-0.62 / -0.63<br>[-0.01]	-0.65 / -0.62<br>[0.03]
T	-0.21 / -0.22<br>[-0.01]	-0.22 / -0.18<br>[0.04]	-0.03 / -0.09<br>[-0.06]
Ti – T	1.02 / 0.99<br>[0.03]	1.05 / 0.97<br>[0.08]	0.70 / 0.72<br>[-0.02]

Table S 2: Average Lowedin charges calculated for different $Ti_2B_2T_2$ ml/ss-MBenes.

The difference between ml- and ss- values is showed inside squared brackets.

References

[1] K. A. Mäder, A. Zunger, *Phys. Rev. B* **1995**, *51*, 10462.

[2] W. Setyawan, S. Curtarolo, *Comput. Mater. Sci.* **2010**, *49*, 299.
